# Supplementary material for: AKT1 phosphorylation of cytoplasmic ME2 induces a metabolic switch to glycolysis for tumorigenesis
Source: Nat Commun. 2024 Jan 23;15:686. doi: 10.1038/s41467-024-44772-8 (PMC10805786; doi:10.1038/s41467-024-44772-8)
Supplement: Supplementary file 1 — Supplementary Information [file 41467_2024_44772_MOESM1_ESM.pdf]

Supplementary Information

AKT1 phosphorylation of cytoplasmic ME2 induces a metabolic switch to glycolysis for tumorigenesis

Taiqi Chen, Siyi Xie , Jie Cheng , Qiao Zhao, Hong Wu, Wenjing Du, Peng Jiang

Supplementary Fig. 1

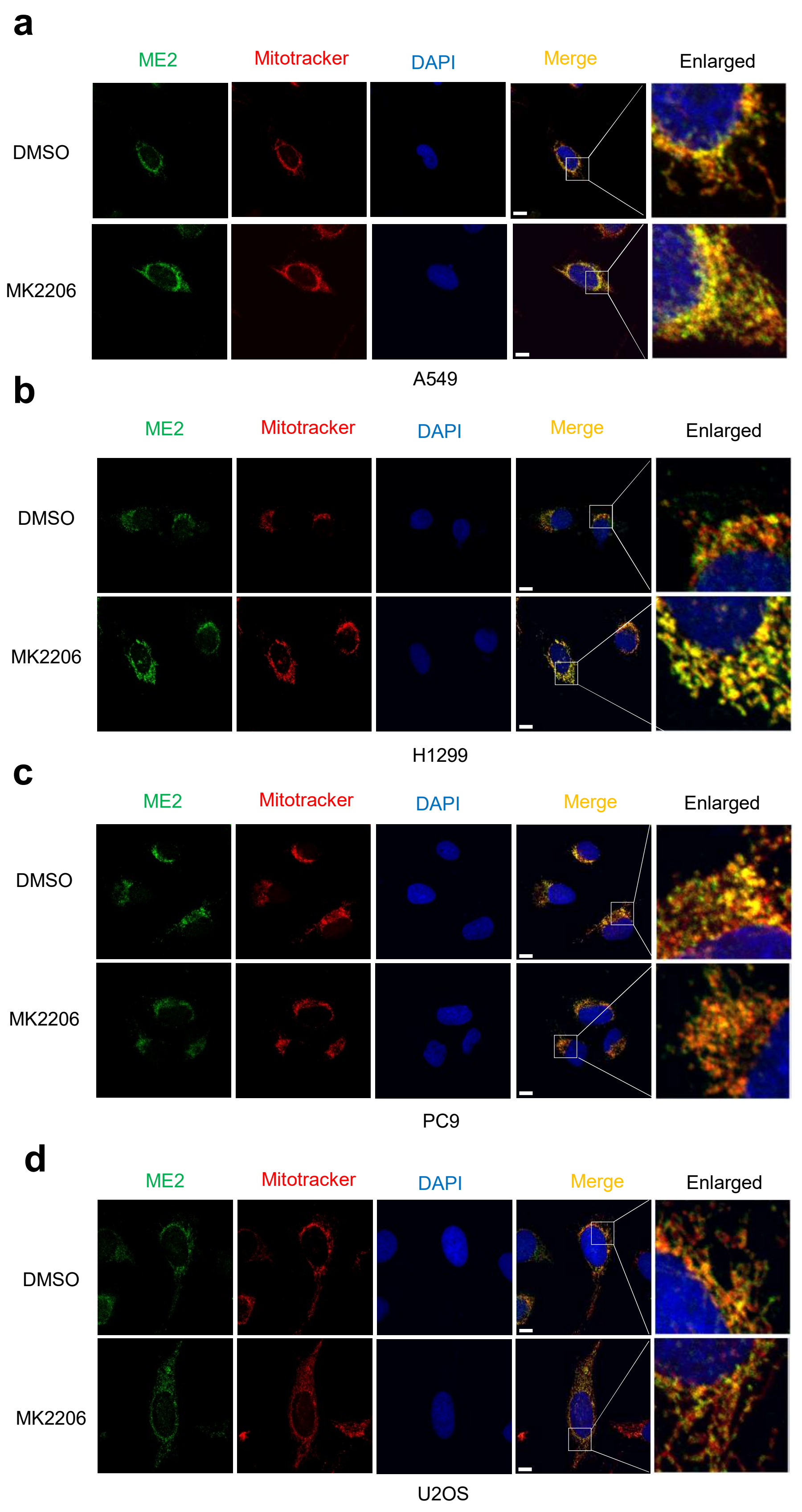

Supplementary Fig. 1 | Effect of the PI3K inhibitor MK2206 on ME2 mitochondrial localization in PTEN-normal expressing tumor cells.

a-d, A549 (**a**), H1299 (**b**), PC9 (**c**) and U2OS (**d**) cells were treated with DMSO or 5 μM MK2206 for 24 hours. Confocal images of subcellular localization of ME2 in these cells were performed after staining with an anti-ME2fl antibody. Scale bars, 10μm.

All data are representative of three independent experiments.

Supplementary Fig. 2

a

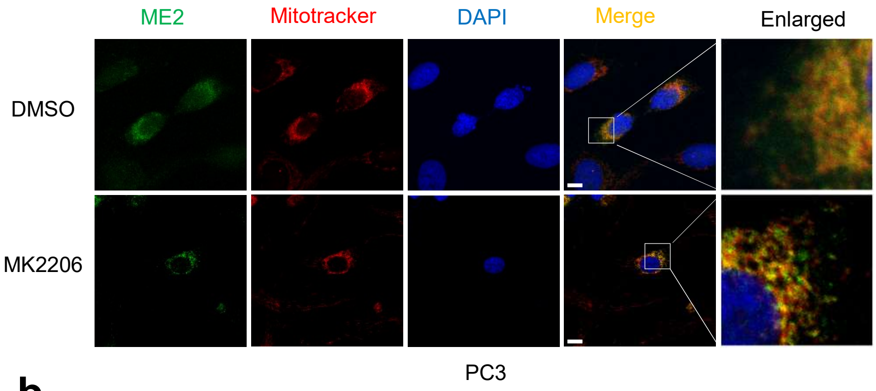

b

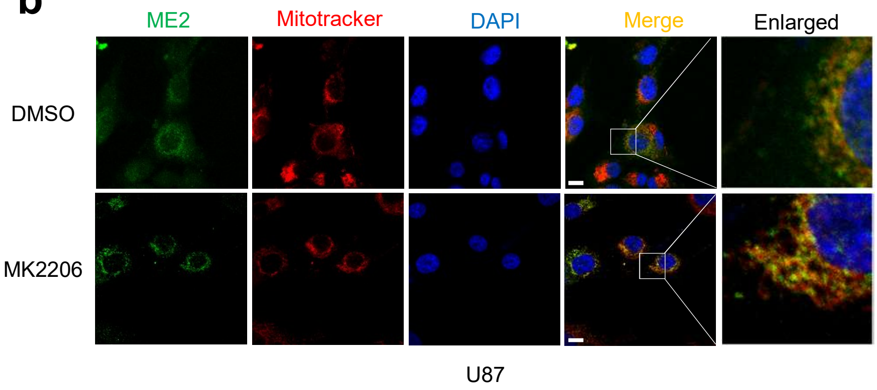

c

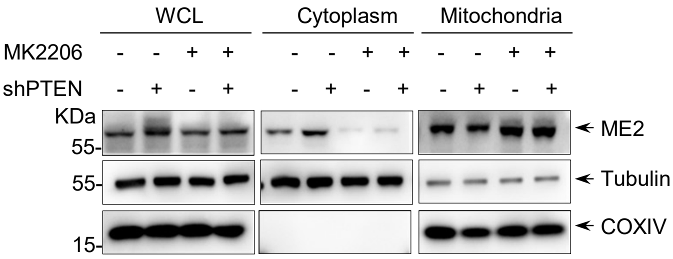

Supplementary Fig. 2 | MK2206 increases mitochondria localization of ME2 even in the absence of PTEN.

a and **b**, PC3 (**a**) and U87 (**b**) cells were treated with DMSO or 5 μM MK2206 for 24 hours. Cells were stained with the anti-ME2fl antibody and confocal images of subcellular localization of ME2 were performed. Scale bars, 10 μm.

c, HepG2 cells stably expressing control shRNA or PTEN shRNA were treated with DMSO (-) or 5 μM MK2206 for 12 hours, followed by fractionation. Each fractionation was analyzed by western blotting using indicated antibodies.

All data are representative of three independent experiments.

Supplementary Fig. 3

a

ME2fl N-terminal sequence

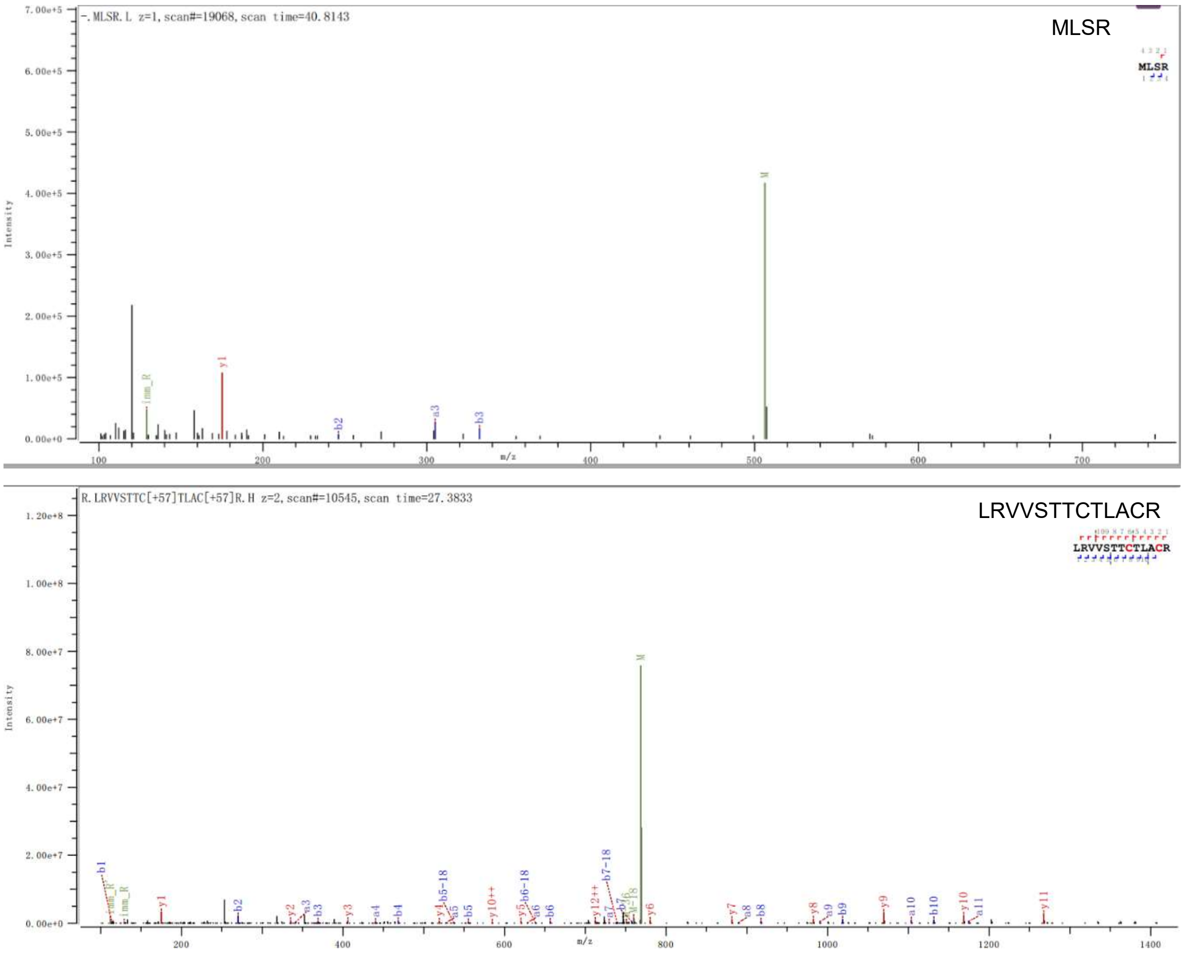

b

ME2 N-terminal sequence

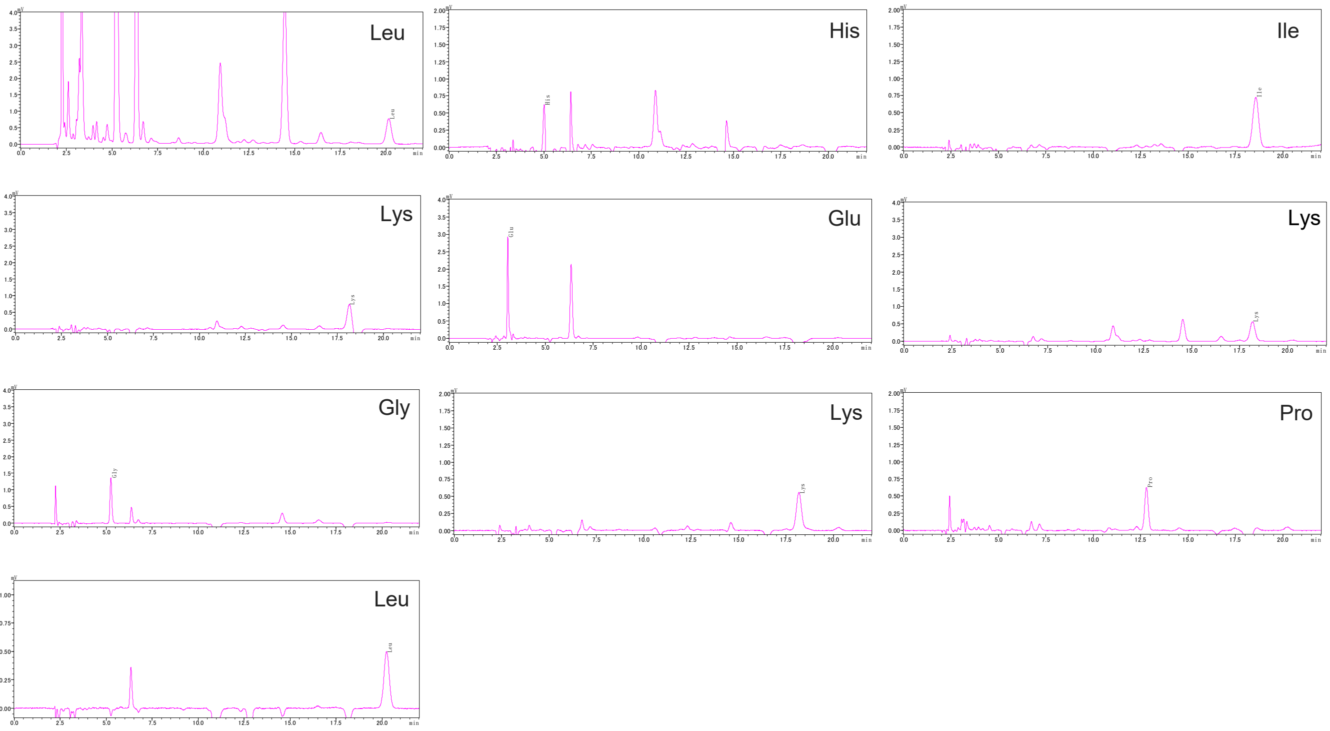

Supplementary Fig. 3 | Mass spectrometry analysis reveals that ME2fl contains an additional 18 AA at the N-terminal compared to ME2.

a, Analysis of the N-terminal amino acid sequence of ME2fl by immunoprecipitation-mass spectrometry. The immunoprecipitated ME2fl-3'Flag proteins from HEK293T cells were transferred to PVDF membrane for mass spectrometry analysis and sequencing of its N-terminal amino acids.

b, Immunoprecipitation-Edman sequencing analysis of the N-terminal amino acid of ME2fl. The immunoprecipitated ME2fl-3'Flag proteins from 293T cells were transferred to PVDF membrane and N-terminal sequencing was performed by sequential Edman reaction. The N-terminal 10 amino acids of ME2fl were sequenced and the position and absorption spectral profile of each sequenced amino acid are shown.

Supplementary Fig. 4

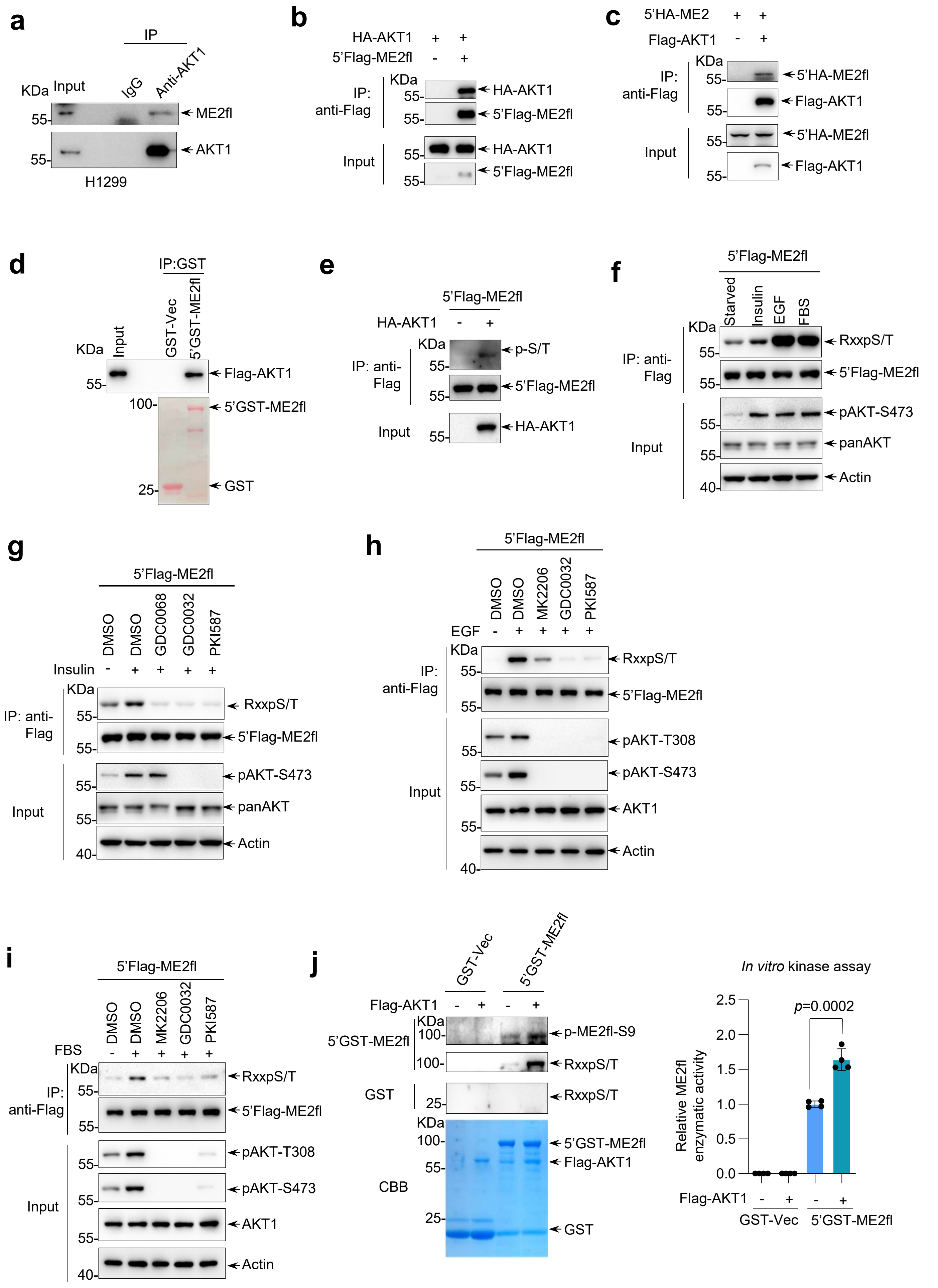

Supplementary Fig. 4 | AKT1 binds to and phosphorylates ME2fl, which is promoted by growth factors.

a, H1299 cells were lysed and immunoprecipitated with the anti-ME2fl antibody or isotype-matching control antibody (IgG). Immunoprecipitants and input were analyzed by western blotting.

b, Lysates of HEK293T cells transfected with plasmids expressing HA-AKT1 together with 5'Flag-ME2fl or vector control for 24 hours were used for immunoprecipitation with an anti-Flag antibody. Immunoprecipitants and input were analyzed by immunoblotting using indicated antibodies.

c, Lysates of 293T cells transfected with 5'HA-ME2fl and Flag-AKT1 or Flag vector control were used for immunoprecipitation with an anti-Flag antibody. Immunoprecipitants were analyzed by immunoblotting.

d, HEK293T purified recombinant Flag-AKT1 was incubated separately with bacterially purified control GST protein or recombinant 5'GST-ME2fl protein conjugated with glutathione-conjugated agarose beads. Bound and input proteins were analyzed by western blotting.

e, Whole cell lysates or anti-Flag immunoprecipitants from transfected 293T cells expressing HA-tagged AKT1 together with 5'Flag-ME2fl were analyzed by immunoblotting. ME2fl phosphorylation was determined by using an anti-phospho-Ser/Thr (pS/T) antibody.

f, HEK293T cells transfected with 5'Flag-ME2fl were serum starved for 24 hours and stimulated with the indicated growth factors for 30 min. Whole-cell lysates (input) and anti-Flag immunoprecipitants were analyzed by western blotting for ME2fl phosphorylation using an anti-RXXpS/T antibody.

g-**i**, HEK293T cells transfected expressing 5'Flag-ME2fl were serum starved for 24 hours and then treated without (-) or with different PI3K inhibitors for 4 hours before stimulation by insulin (**g**), EGF (**h**) or FBS (**i**) for another 30 min as indicated. Cells were immunoprecipitated with an anti-Flag antibody and analyzed by western blotting using the indicated antibodies. ME2fl phosphorylation was determined by immunoblotting using an anti-RXXpS/T antibody.

j, Purified recombinant 5'GST-ME2fl and GST proteins were incubated with Flag-AKT1 *in vitro* as indicated in the presence of ATP for 30 min. ME2fl phosphorylation was determined by immunoblotting using an anti-RXXpS/T and an anti-pME2flS9 antibodies respectively. The enzymatic activity of the recombinant GST-ME2fl was measured (n=4 for each group). Data are means ± SD; two-tailed Student's t test. All data are representative of three independent experiments.

Supplementary Fig. 5

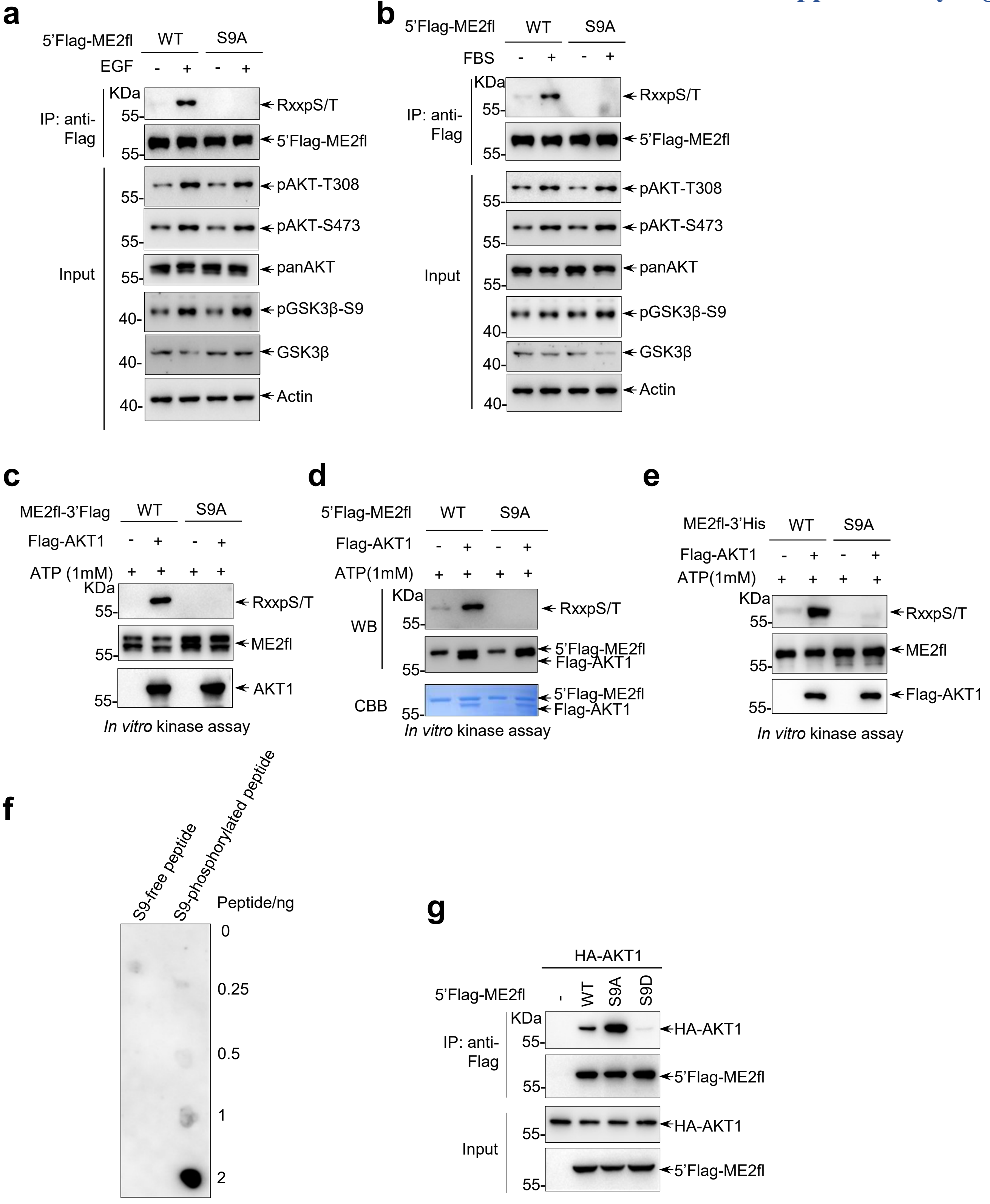

Supplementary Fig. 5 | Identification of serine 9 on ME2fl as a phosphorylation target of AKT1.

a and **b**, HEK293T cells expressing 5'Flag-ME2fl (WT) or 5'Flag-ME2flS9A were serum starved for 24 hours and then cultured with 50 ng/mL EGF(**a**) or 10% FBS(**b**) for another 30 min. Whole-cell lysates and anti-Flag immunoprecipitants were analyzed by western blot for ME2fl phosphorylation using an anti-RXXpS/T antibody and expression of the indicated proteins.

c-e, Purified recombinant ME2fl-3'Flag and ME2flS9A-3'Flag (**c**), 5'Flag-ME2fl and 5'Flag-ME2flS9A(**d**), or ME2fl-3'His and ME2flS9A-3'His (**e**) proteins were incubated with Flag-AKT1 protein *in vitro* in the presence of ATP for 30 min. ME2fl phosphorylation was determined by immunoblotting using an anti-RXXpS/T antibody.

f, Generation of the rabbit polyclonal antibodies against ME2fl-phosphorylated S9 (p-ME2fl). Peptide of SRLRVV(S-p)TT, modified with phosphorylated S9 and coupled to KLH, was used to generate the antibodies, which were validated by dot-blotting with control peptides or phosphorylated peptides.

g, Lysates of HEK293T cells transfected with HA-AKT1 together with 5'Flag-ME2fl, 5'Flag-ME2flS9A, 5'FLAG-ME2flS9D or FLAG vector control (-) were used for immunoprecipitation with an anti-Flag antibody. Immunoprecipitants were analyzed by immunoblotting. All immunoblotting data are representative of three independent experiments.

Supplementary Fig. 6

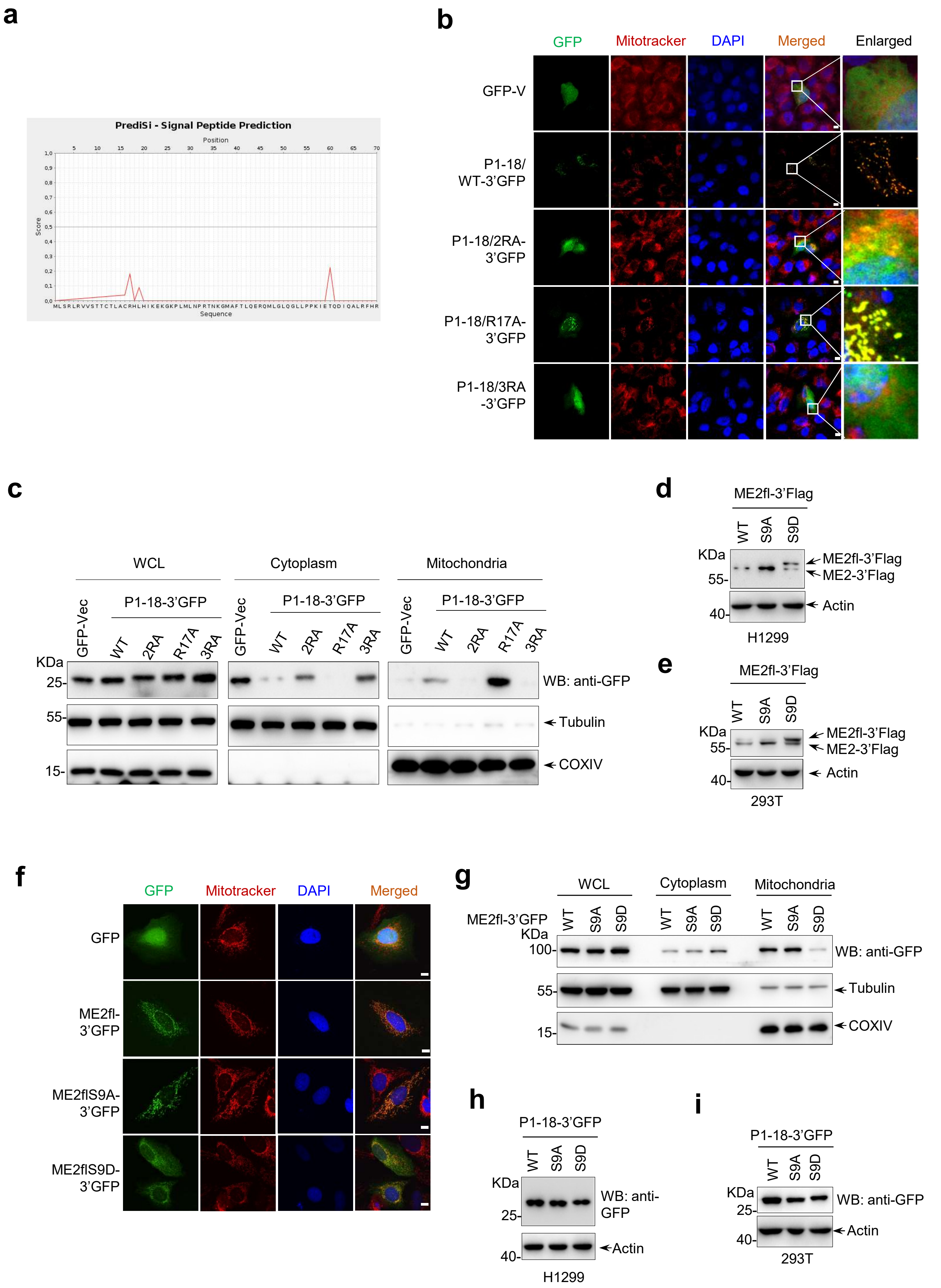

Supplementary Fig. 6 | The N-terminal 1-18AA of ME2fl is a mitochondrial targeting signal (MTS) peptide, whose function is inhibited by Ser9 phosphorylation.

a, Signal peptide prediction using Predisi (www.predisi.de) for the N-terminal of ME2fl. Shown is the predicted mitochondrial target signal (MTS) in the N-terminal 70 amino acids (AA) of ME2fl.

b and **c**, Confocal image (**b**) and cell fractionation (**c**) analysis of ME2fl Mitochondrial Targeting Signal (MTS). U2OS cells expressing 3'GFP-tagged wild-type (WT) 1-18AA peptide (P1-18/WT-3'GFP) or 1-18AA peptide carrying different point mutation(s) as indicated (P1-18/2RA-3'GFP, P1-18/R17A-3'GFP and P1-18/3RA-3'GFP) were analyzed by confocal imaging for GFP localization (**b**) and cell fractionation followed by immunoblotting. P1-18, the N-terminal 1-18 AA of ME2fl; WT, Wild Type; 2RA, R4AR6A; 3RA, R4AR6AR17A. Scale bars, 10 μm.

d and **e**, Lysates of H1299 cells (**d**) and HEK293T cells (**e**) transfected with 3'Flag-tagged ME2fl (ME2fl-3'Flag), ME2flS9A-3'Flag or ME2flS9D-3'Flag were analyzed by western blotting with an anti-Flag antibody.

f, Subcellular localization analysis of U2OS cells expressing 3'GFP-tagged ME2fl (ME2fl-3'GFP), ME2flS9A-3'GFP, ME2flS9D-3'GFP, or GFP vector control. Scale bars, 10 μm.

g, HEK293T cells transfected with ME2fl-3'GFP, ME2flS9A-3'GFP, or ME2flS9D-3'GFP for 24 hours were subcellularly fractionated and analyzed by immunoblotting using an anti-GFP antibody. β-tubulin and COXIV served as loading controls as well as cytosolic and mitochondrial markers, respectively.

h and **i**, Cell lysates of H1299 cells (**h**) and HEK293T cells (**i**) expressing 3'GFP tagged 1-18AA peptide of ME2fl (P1-18/WT-3'GFP), or 1-18AA peptide carrying S9A or S9D as indicated (P1-18/S9A-3'GFP and P1-18/S9D-3'GFP) were analyzed by immunoblotting with an anti-GFP antibody.

All data are representative of three independent experiments.

Supplementary Fig. 7

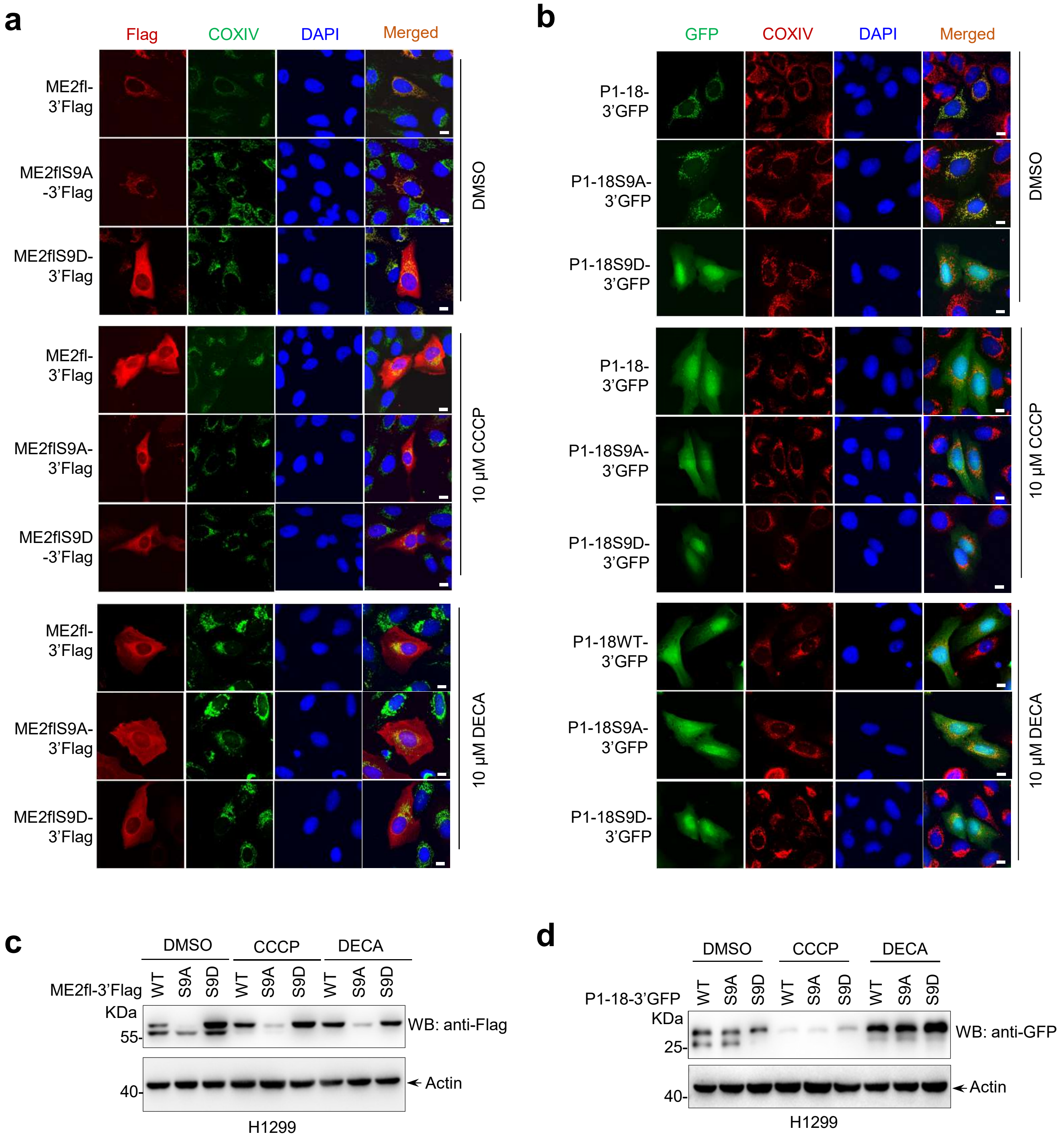

Supplementary Fig. 7 | Phosphorylation of 1-18AA at Ser 9 determines the cytoplasmic localization of ME2fl.

a, U2OS cells expressing 3'Flag-tagged ME2fl (ME2fl-3'Flag), ME2flS9A-3'Flag, or ME2flS9A-3'Flag were treated with 10 µM CCCP, 10 µM DECA, or DMSO for 24 hours, followed by immunofluorescence analysis. DECA, Dequalinium chloride; CCCP, Carbonyl cyanide-m-chlorophenylhydrazone. Scale bars, 10 µm.

b, U2OS cells expressing 3'GFP-tagged 1-18AA peptide of ME2fl (P1-18/WT-3'GFP), or 1-18AA peptide carrying S9A or S9D as indicated (P1-18/S9A-3'GFP and P1-18/S9D-3'GFP) were treated with 10 µM CCCP, 10 µM DECA, or DMSO. 24 hours later, immunofluorescence analysis of the cells was performed. Scale bars, 10 µm.

c, H1299 cells expressing 3'Flag-tagged ME2fl (ME2fl-3'Flag), ME2flS9A-3'Flag, ME2flS9A-3'Flag were treated with 10 µM CCCP, 10 µM DECA, or DMSO as indicated for 24 hours. The cells were then lysed and analyzed by western blotting with an anti-Flag antibody.

d, H1299 cells expressing 3'GFP-tagged 1-18AA peptide of ME2fl (P1-18/WT-3'GFP), or 1-18AA peptide carrying S9A or S9D as indicated (P1-18/S9A-3'GFP and P1-18/S9D-3'GFP) were treated with 10 µM CCCP, 10 µM DECA, or DMSO as indicated for 24 hours, followed by immunoblotting with an anti-GFP antibody. All immunoblotting data are representative of three independent experiments.

Supplementary Fig. 8

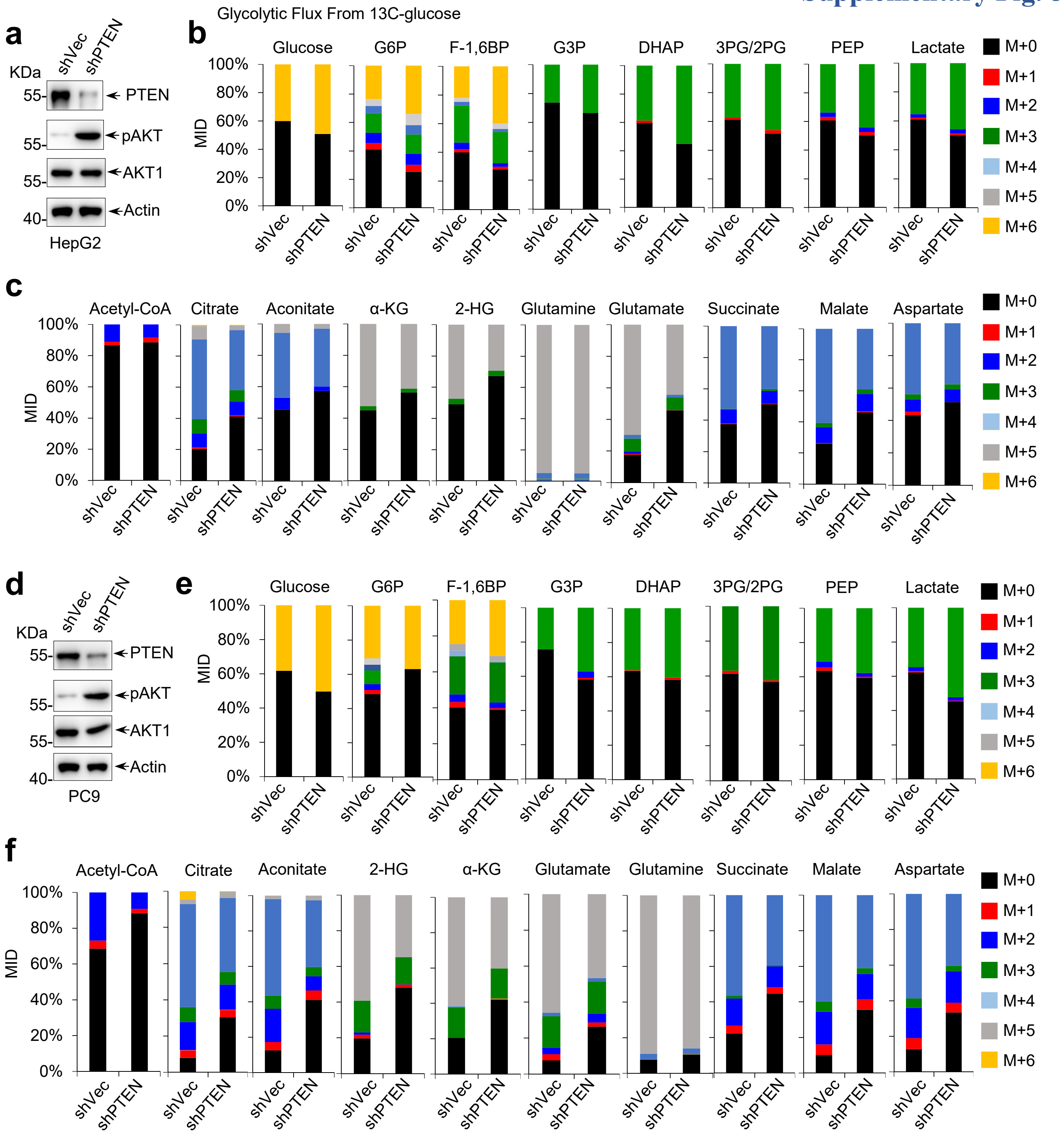

Supplementary Fig. 8 | Effect of PTEN depletion on glycolysis and TCA cycle.
a-c, HepG2 cells expressing PTEN-Targeting shRNA or control shRNA were cultured with [U-$^{13}C_6$]glucose or [U-$^{13}C_5$]glutamine for 6 hours. Protein expression was analyzed by western blotting with indicated antibodies (**a**). Mass isotopomer distribution (MID) of ^{13}C-labeled glycolytic intermediates derived from [U-$^{13}C_6$]glucose (**b**), and MID of ^{13}C-labeled TCA cycle intermediates derived from [U-$^{13}C_5$]glutamine (**c**) were determined by LC-MS analysis respectively.
d-f, PC9 cells expressing PTEN-Targeting shRNA or control shRNA were cultured with [U-$^{13}C_6$]glucose or [U-$^{13}C_5$]glutamine for 6 hours. Protein expression was analyzed by western blotting with the indicated antibodies (**d**). The ^{13}C mass isotopomer distribution (MID) of glycolytic intermediates derived from [U-$^{13}C_6$]glucose (**e**), and the TCA cycle intermediates derived from [U-$^{13}C_5$]glutamine were determined (**f**) by LC-MS analysis respectively.

Supplementary Fig. 9

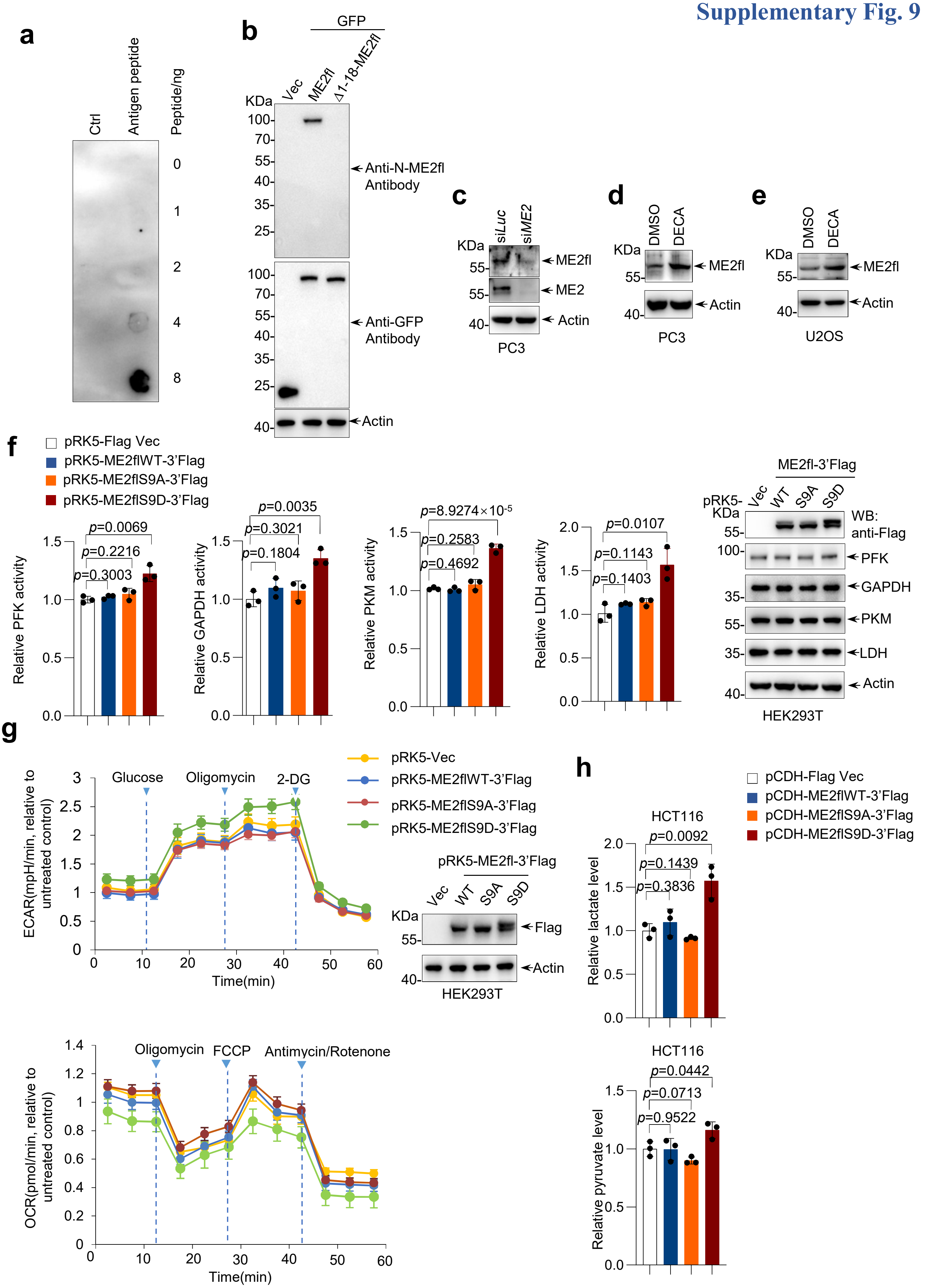

Supplementary Fig. 9 | ME2fl phosphorylation by AKT1 promotes glycolysis.

a, Production of rabbit polyclonal antibodies against 1-18 amino acids of ME2fl (anti-ME2fl). Peptide of SRLRVVSTTCTLACRH coupled to KLH, was used to generate the antibodies and the antibodies were validated by dot-blotting with control peptides or ME2fl 1-18 amino acids peptides.

b, Lysates of HEK293T cells expressing GFP vector (GFP-Vec), 5'GFP-tagged ME2fl (5'GFP-ME2fl) or 5'GFP-ME2fl-Δ1-18AA were analyzed with the anti-ME2fl antibody and an anti-GFP antibody, respectively.

c and **d**, Lysates of PC3 cells transfected with ME2fl siRNA or control siRNA(**c**), or treated with DMSO or 10μM DECA (**d**) were analyzed by immunoblotting with the the anti-ME2fl antibody.

e, U2OS cells treated with DMSO or 10μM DECA for 24 hours were analyzed by western blotting using the anti-ME2fl antibody.

f, HEK293T cells transfected with pRK5-ME2fl-3'Flag, pRK5-ME2flS9A-3'Flag, pRK5-ME2flS9D-3'Flag, or pRK5-Flag vector control were lysed and subjected to glycolytic enzymatic assays (left, n=3 biologically independent experimental repeats for each group). Protein expression was determined by western blot analysis (right). Data are means ± SD; two-tailed Student's t test.

g, ECAR or OCR analysis of HEK293T cells transfected with the indicated plasmids as in (**f**). For ECAR analysis, 25 mM glucose, 1 μM oligomycin, and 100 mM 2-DG were supplied at the indicated times (n=8 biologically independent experimental repeats for each group); For OCR analysis, 1 μM oligomycin, 1 μM FCCP and 2.5μM antimycin/rotenone were supplied at the indicated times (n=8 biologically independent experimental repeats for each group). ECAR and OCR were measured using Seahorse XFe96 analyzer. Protein expression in transfected HEK293T was analyzed by western blotting (right). All immunoblotting data are representative of three independent experiments. Data are means ± SD; *P< 0.05; **P< 0.01; ***P< 0.001; ****P< 0.0001; NS, no significance; two-tailed Student's t test. Exact P values are shown in Source data.

h, Relative lactate and pyruvate levels in HCT116 cells stably expressing ME2fl (pCDH-ME2fl -3'Flag), ME2flS9A (pCDH-ME2flS9A-3'Flag), ME2flS9D (pCDH-ME2flS9D-3'Flag), or vector control (pCDH-Flag Vec) were measured by LC-MS (n=3 biologically independent experimental repeats for each group). Data are means ± SD; two-tailed Student's t test.

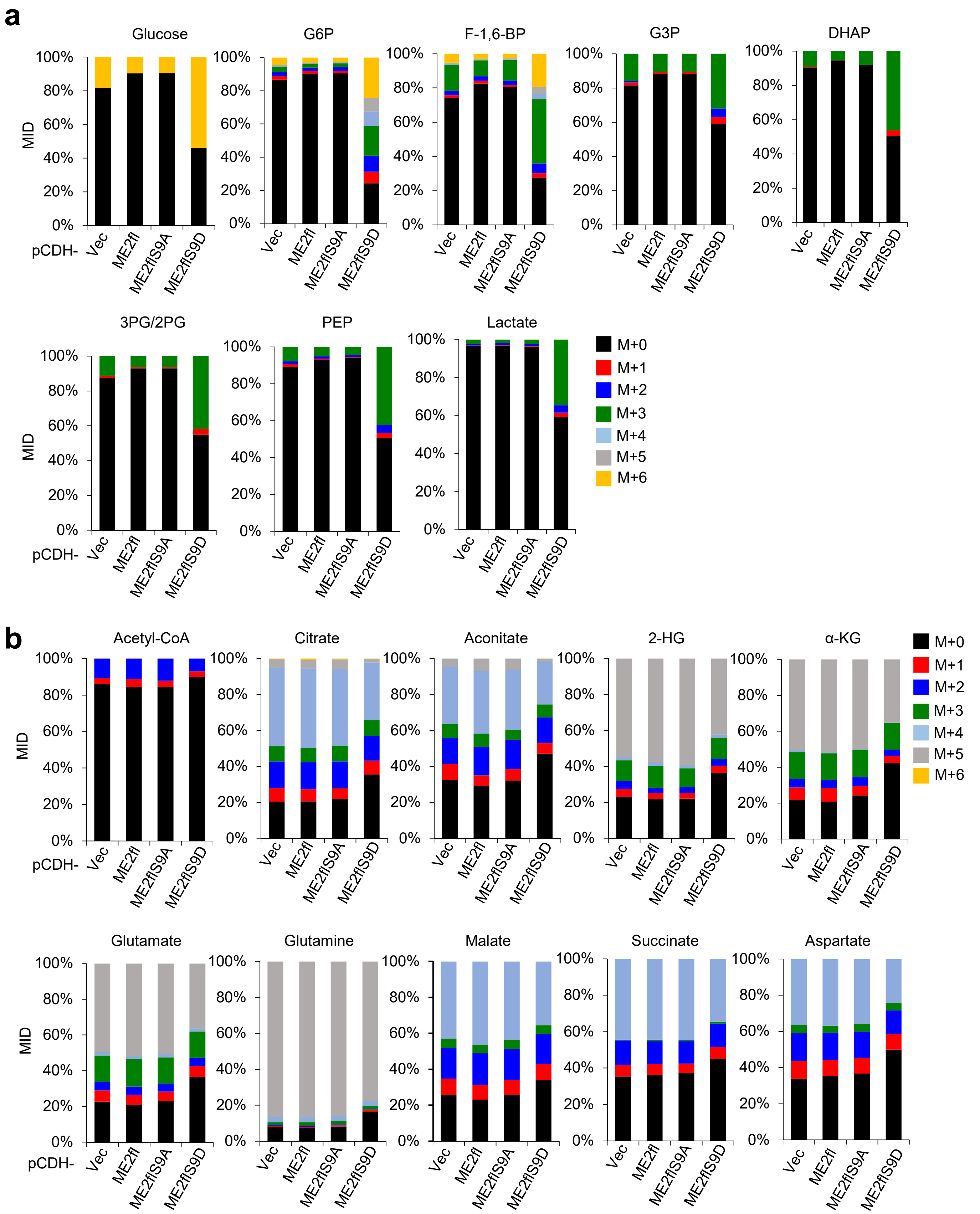
a
Glucose
G6P
F-1,6-BP
G3P
DHAP
3PG/2PG
PEP
Lactate
MID
pCDH-
Vec
ME2fl
ME2flS9A
ME2flS9D
0%
20%
40%
60%
80%
100%
M+0
M+1
M+2
M+3
M+4
M+5
M+6
b
Acetyl-CoA
Citrate
Aconitate
2-HG
α-KG
Glutamate
Glutamine
Malate
Succinate
Aspartate

Supplementary Fig. 10 | The S9D mutation confers on ME2fl the ability to promote glycolysis.

a and **b**, HCT116 cells stably expressing wild-type ME2fl (pCDH-ME2fl-3'Flag), ME2flS9A (pCDH-ME2flS9A-3'Flag), ME2flS9D (pCDH-ME2flS9D-3'Flag), or vector control (pCDH-Flag Vec) were cultured with [U-$^{13}C_6$]glucose or [U-$^{13}C_5$]glutamine for 6 hours. Mass isotopomer distribution (MID) of ^{13}C-labeled glycolytic intermediates derived from [U-$^{13}C_6$]glucose (**a**), and MID of ^{13}C-labeled TCA cycle intermediates derived from [U-$^{13}C_5$]glutamine (**c**) were determined by LC-MS analysis respectively. Data were verified in at least three independent experiments.

Supplementary Fig. 11

a

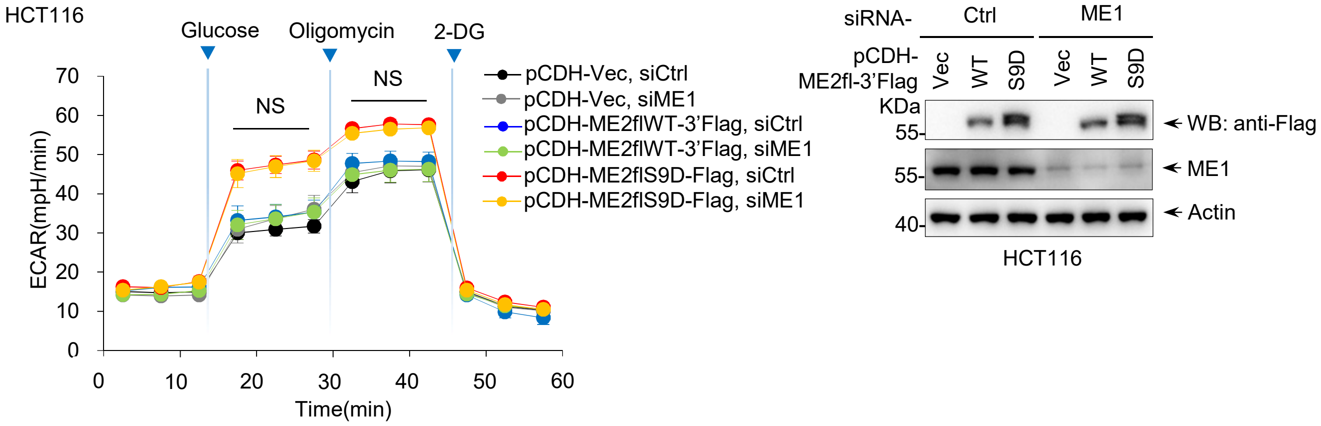

b

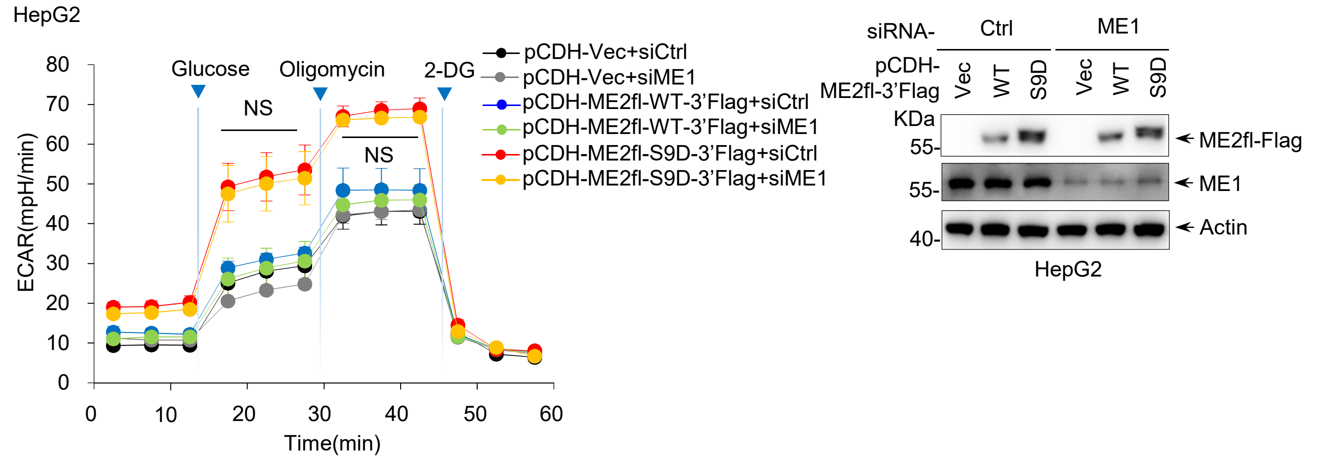

Supplementary Fig. 11 | ME1 does not affect ME2fl-mediated promotion of glycolysis.
a, HCT116 cells stably expressing wild-type ME2fl (pCDH-ME2fl-3'Flag), ME2flS9D (pCDH-ME2flS9D-3'Flag), or vector control (pCDH-Flag Vec) were transfected with siRNA targeting ME1 or control siRNA for 48 hours. Transfected cells were treated with 25 mM glucose, 1 μM oligomycin and 100 mM 2-DG at the indicated times for ECAR analysis in a Seahorse XFe96 analyzer (n=6 for each group). Protein expression was analyzed by western blotting with indicated antibodies. Data are means ± SD; NS, no significance; two-tailed Student's t test. Exact *P* values are shown in Source data.

b, HepG2 cells stably expressing wild-type ME2fl (pCDH-ME2fl-3'Flag), ME2flS9D (pCDH-ME2flS9D-3'Flag), or vector control (pCDH-Flag Vec) were transfected with siRNA targeting ME1 or control siRNA for 48 hours. ECAR of the transfected cells were analyzed in a Seahorse XFe96 analyzer (n=6 for each group). Protein expression was determined by western blot analysis using indicated antibodies. Data are means ± SD; NS, no significance; two-tailed Student's t test. Exact *P* values are shown in Source data.

Supplementary Fig. 12

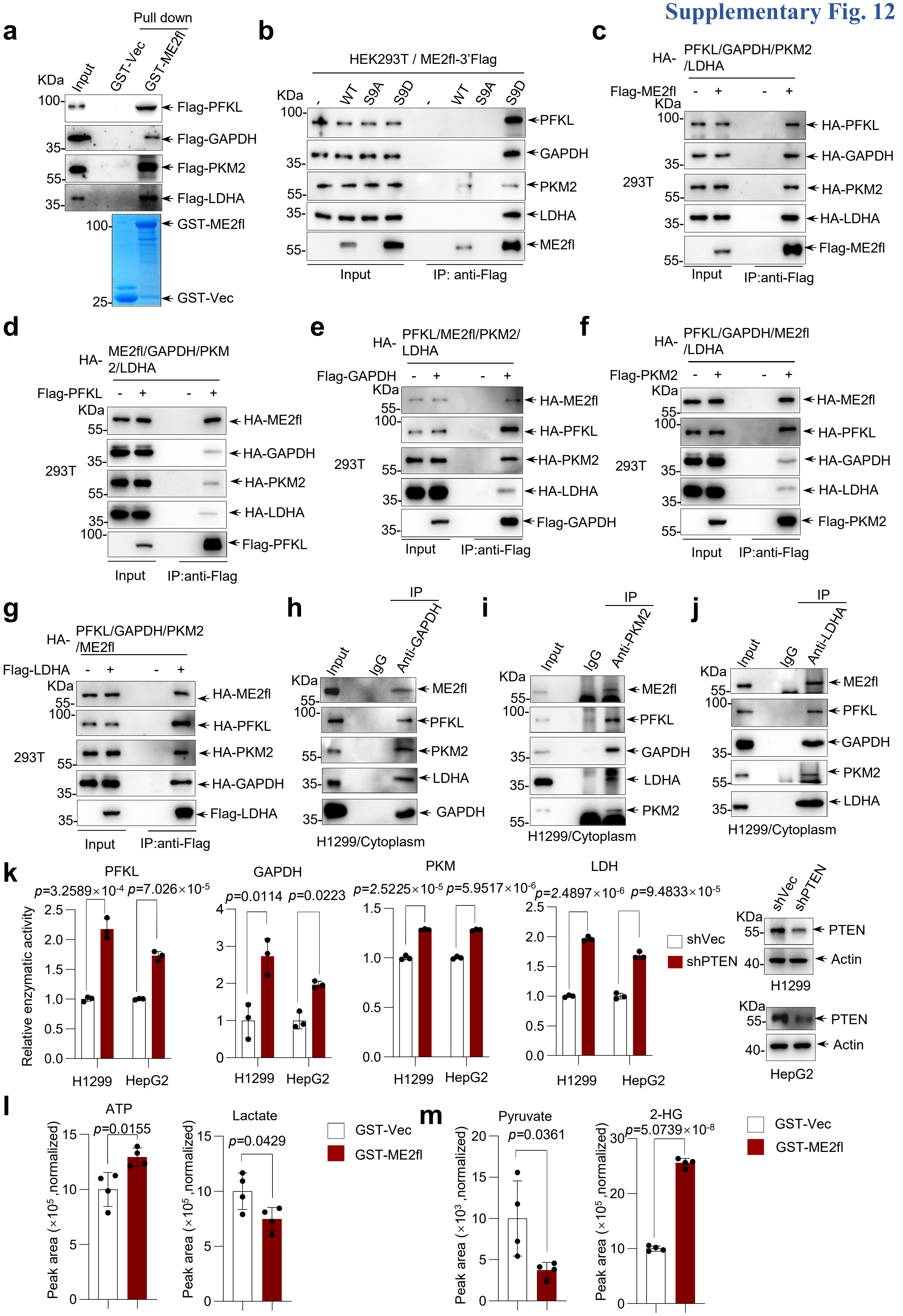

Supplementary Fig. 12 | ME2fl forms an activation complex with glycolytic enzymes.

a, Purified Flag-tagged PFKL, GAPDH, PKM2 and LDHA were incubated together with control GST or recombinant 5'GST-ME2fl proteins conjugated with glutathione-conjugated agarose beads as indicated. Bound and input proteins were analyzed by western blotting.

b, HEK293T cells expressing ME2fl-3'Flag (WT), ME2flS9A-3'Flag, or ME2flS9D-3'Flag were subcellularly fractionated, immunoprecipitated with an anti-Flag antibody and then analyzed by western blotting using the the anti-PFKL, anti-GAPDH, anti-PKM2 and anti-LDHA antibodies, respectively.

c-g, Reciprocal co-association among PFKL, GAPDH, PKM2, LDHA and ME2fl. Cell lysates from HEK293T cells expressing the indicated plasmids were immunoprecitated with an anti-Flag antibody and immunoprecipitants were analyzed by western blotting.

h-j, Cytoplasmic fractions of H1299 cells were immunoprecipitated with an anti-GAPDH antibody (**h**), an anti-PKM2 antibody (**i**) or an anti-LDHA antibody (**j**) and the immunoprecipitants were analyzed by western blotting with the anti-ME2fl, anti-PFKL, anti-GAPDH, anti-PKM2 and anti-LDHA antibodies, respectively.

k, H1299 cells or HepG2 cells expressing vector control shRNA or shRNA targeting PTEN were lyzed and the activities of PFKL, GAPDH, PKM2 and LDHA were measured (left, n=3 for each group) and shRNA knockdown efficiency was analyzed by western blotting (right).

l and **m**, Purified Flag-tagged PGI, PFKL, ALDOA, TPI, GAPDH, PGK, PGAM, ENO, PKM2 and LDHA were incubated in the glycolytic assay buffer containing 50 mM Tris-HCl, 15 mM $MgCl_2$, 2 mM K_3PO_4, 1 mM G6P, 1 mM ATP, 1 mM ADP, 1 mM NAD^+, and 1 mM NADH in the absence or presence of control GST-Vector or GST-ME2fl as indicated. The production of ATP, lactate, pyruvate and 2-HG was measured by LC-MS. n=4 for each group in **l** and **m**.

Data are means ± SD; two-tailed Student's t test. All immunoblotting data are representative of three independent experiments.

Supplementary Fig. 13

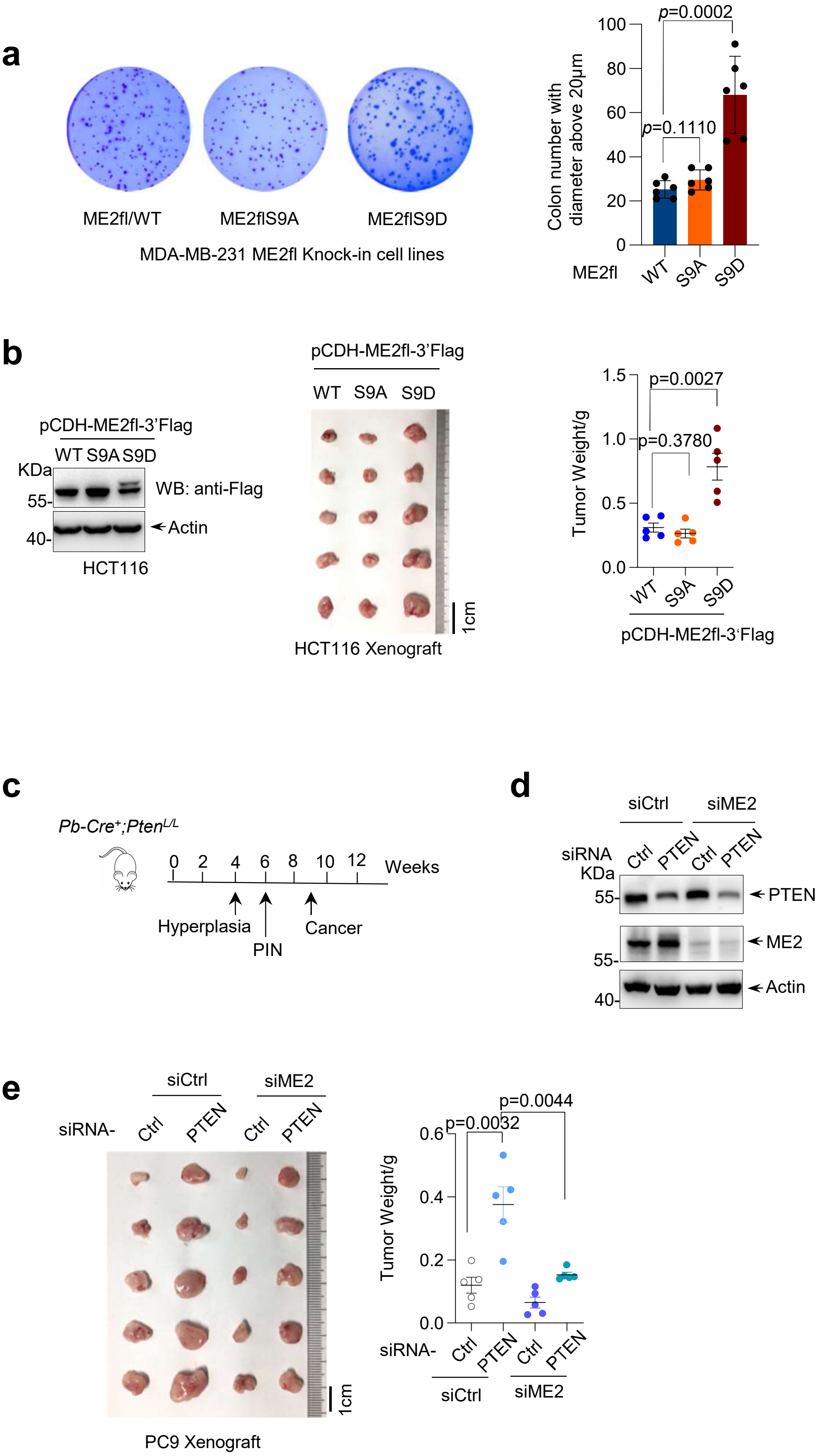

Supplementary Fig. 13 | S9D mutation enhances the ability of ME2 to promote tumor growth.

a, Colony formation assay of MDA-MB-231 cells with knock-in expression of wild-type ME2fl (WT), ME2flS9A, or ME2flS9D. Numbers of colonies with a diameter above 20 μm were quantified (n=6 biologically independent experimental repeats for each group). Data are means ± SD; two-tailed Student's t test.

b, HCT116 cells stably expressing ME2fl-3'Flag, ME2flS9A-3'Flag or ME2flS9D-3'Flag were injected subcutaneously into nude mice. Mice were analyzed for tumor formation and tumor weight was calculated after two weeks (n=5 mice for each group). ME2fl expression was analyzed by western blotting using an anti-Flag antibody. Data are means ± SD; two-tailed Student's t test.

c, Schematic representation of prostate tumor progression in *Pten*-conditional knockout mice.

d and **e**, PC9 cells transfected with control siRNA, PTEN siRNA and/or ME2 siRNA were subcutaneously injected into nude mice. Tumors were photographed and weighted two weeks later (n=5 mice for each group). Data are means ± SD; two-tailed Student's t test. All immunoblotting data are representative of three independent experiments.

Supplementary Fig. 14

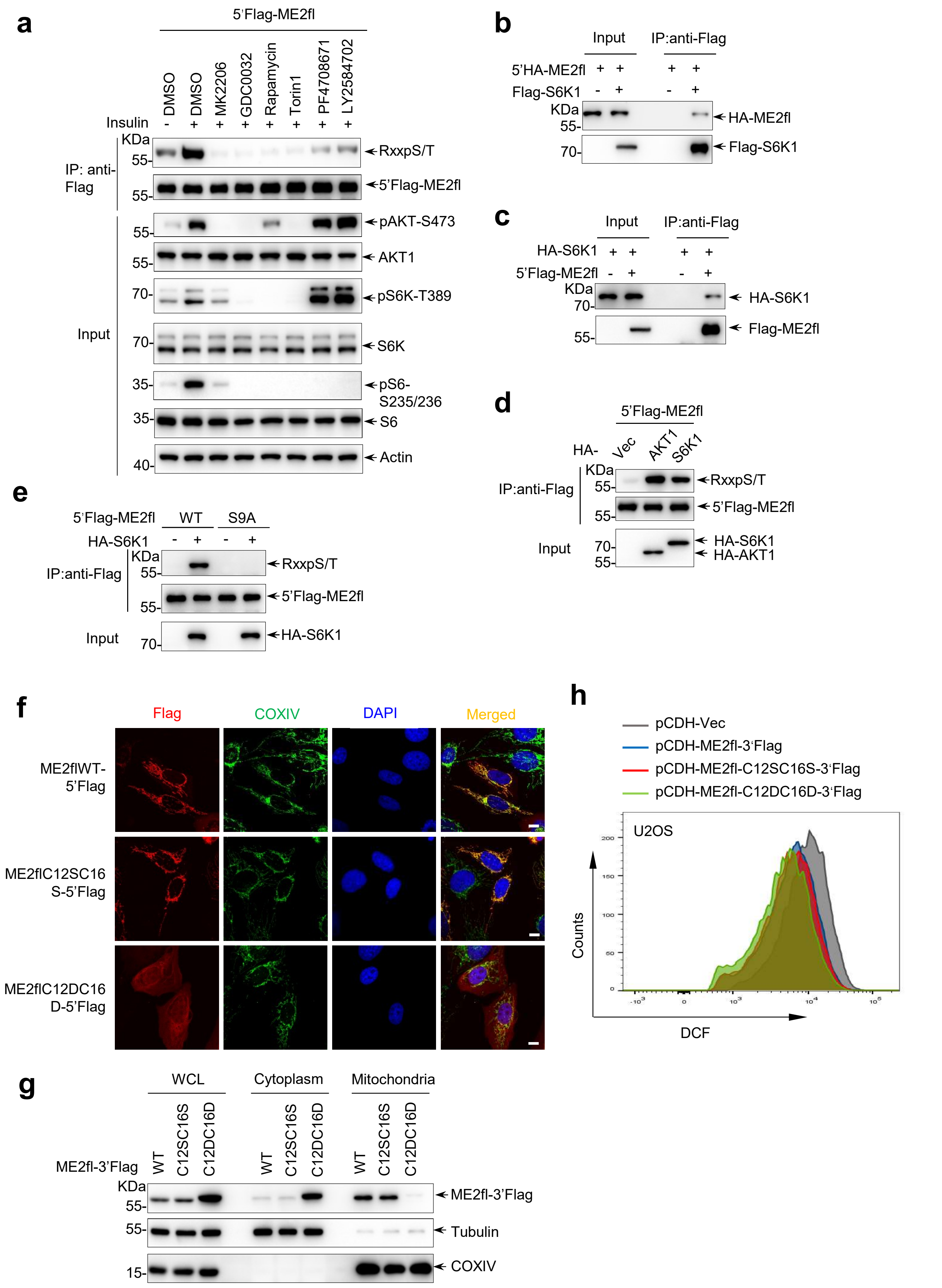

Supplementary Fig. 14 | Ser 9 on ME2fl is also a phosphorylation site for S6K1, and oxidation of cysteines 12 and 16 may influence the entry of ME2 into mitochondria.

a, HEK293T cells transfected with 5'Flag-ME2fl were serum-starved for 24 hours, then treated with DMSO (-), PI3K/AKT inhibitors (MK2206, GDC0032), mTOR inhibitors (Rapamycin, Torin1), or S6K inhibitors (PF4708671, LY2584702) as indicated for 4 hours, followed by insulin stimulation for another 30 min. Cells were immunoprecipitated with an anti-Flag antibody and analyzed by western blotting using indicated antibodies. ME2fl phosphorylation was determined by immunoblotting using an anti-RXXpS/T antibody.

b, Total lysates or anti-Flag immunoprecipitants from transfected HEK293T cells expressing HA-tagged ME2fl (HA-ME2fl) together with Flag-tagged S6K1 or Flag vector control as indicated were analyzed by immunoblotting.

c, Total lysates or anti-Flag immunoprecipitants from transfected HEK293T cells expressing HA-tagged S6K1 together with 5'Flag-tagged ME2fl or Flag vector control as indicated were analyzed by immunoblotting.

d, HEK293T cells were transfected with plasmids expressing 5'Flag-tagged ME2fl (5'Flag-ME2fl) and HA-tagged AKT1 or S6K1 for 24 hours and immunoprecipitated with an anti-Flag antibody. Whole cell lysates (input) and immunoprecipitants were analyzed by immunoblotting using an anti-RXXpS/T antibody.

e, Lysates from HEK293T cells transfected with 5'Flag-ME2fl (WT) or ME2fl dephosphomimic mutant ME2flS9A (5'Flag-ME2flS9A) together with HA-S6K1 or vector control were immunoprecipitated with an anti-Flag antibody and phosphorylation of ME2fl was analyzed by immunoblotting.

f. Immunofluorescence analysis of ME2 in U2OS cells expressing wild-type ME2fl (ME2fl-3'Flag), ME2-C12SC16S-3'Flag and ME2fl-C12DC16D-3'Flag using an anti-Flag antibody. Mitochondria were stained with an anti-COXIV antibody and DNA was stained with DAPI. Scale bars,10μm.

g. HEK293T cells were transfected with ME2fl-3'Flag, ME2-C12SC16S-3'Flag or ME2fl-C12DC16D-3'Flag as indicated, followed by fractionation. Fractionations were analyzed by western blot using indicated antibodies. β-tubulin and COXIV were used as loading controls and as cytosolic and mitochondrial markers, respectively.

h, ROS levels were determined by 2',7'-dichlorodihydrofluorescein diacetate (DCF) in U2OS cells stably expressing wild-type ME2fl (pCDH-ME2fl-3'Flag), ME2flC12SC16S (pCDH-ME2flC12SC16S-3'Flag), ME2flS9D (pCDH-ME2flC12DC16D-3'Flag) or vector control (pCDH-Flag Vec).

Supplementary Fig. 15

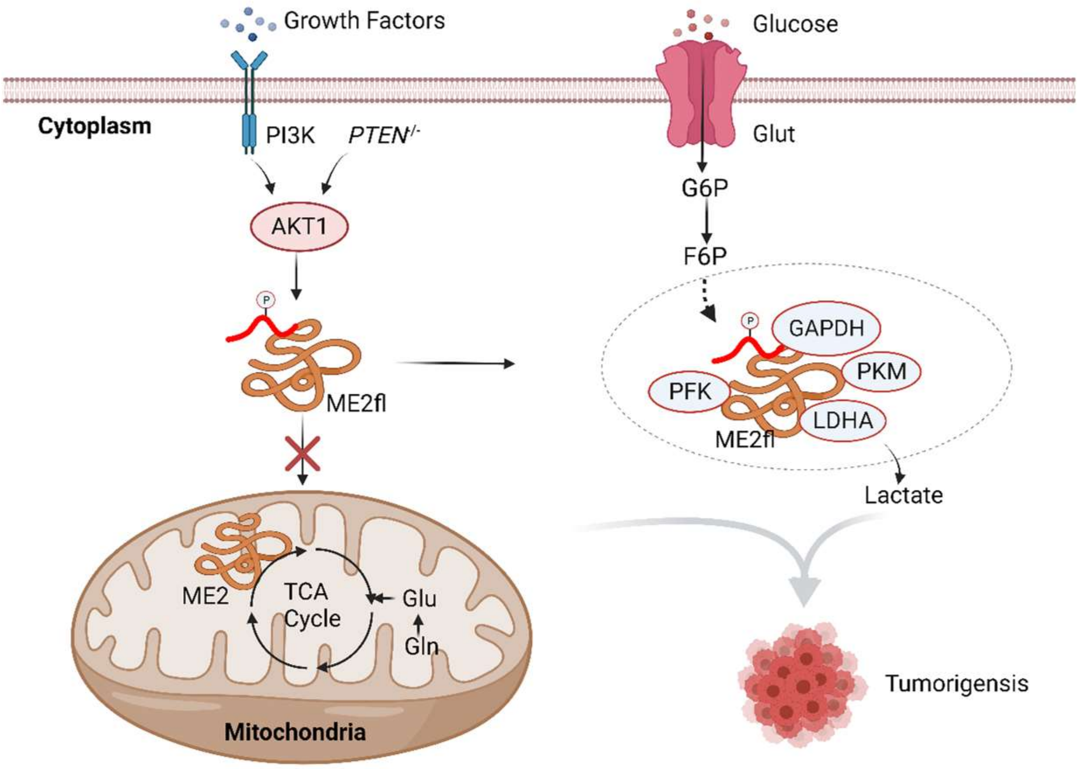

Supplementary Fig. 15 | A working model illustrates how PTEN loss or AKT1 activation phosphorylates ME2fl and induces a metabolic switch towards glycolysis for tumorigenesis. In response to growth factor stimulation or PTEN loss, a cytoplasmic ME2 (ME2fl) is directly phosphorylated by AKT1 at ser 9, leading to cytoplasmic anchoring of ME2fl and enhancing its enzymatic activity. Moreover, by bridging multiple key glycolytic enzymes together, ME2fl promotes glycolytic flux to fuel tumorigenesis.

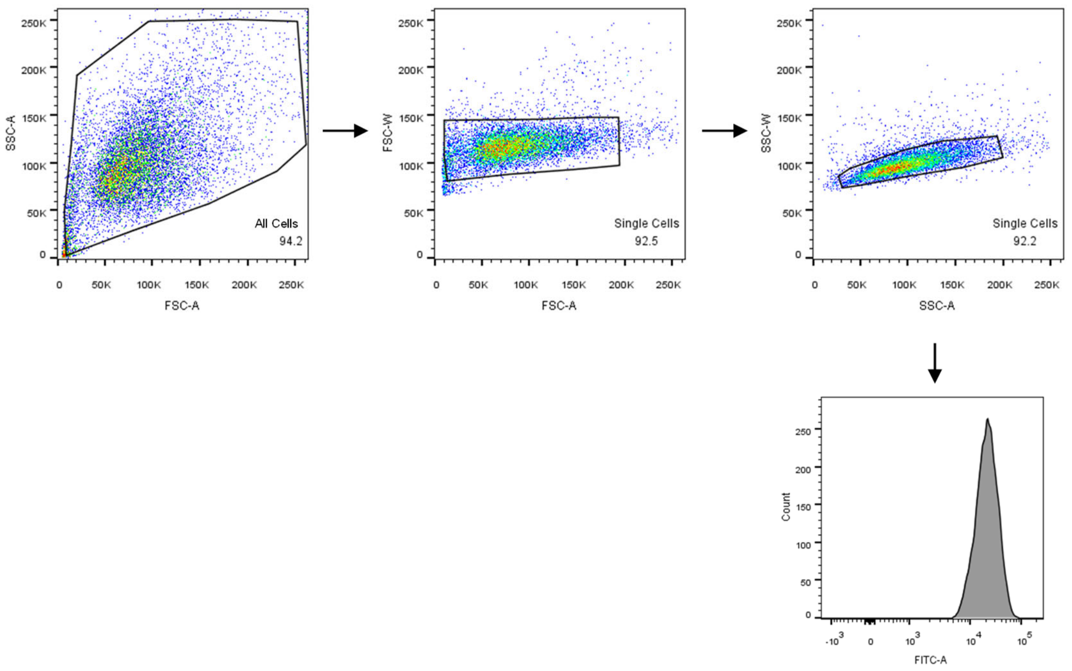

Supplementary Information | Gating strategies used for FACS analysis of ROS.
