# Supplementary material for: AKT1 phosphorylation of cytoplasmic ME2 induces a metabolic switch to glycolysis for tumorigenesis
Source: Nat Commun. 2024 Jan 23;15:686. doi: 10.1038/s41467-024-44772-8 (PMC10805786; doi:10.1038/s41467-024-44772-8)
Supplement: Supplementary file 2 — Reporting Summary [file 41467_2024_44772_MOESM2_ESM.pdf]

Corresponding author(s): Peng Jiang

Last updated by author(s): Nov 21, 2023

Reporting Summary

Nature Portfolio wishes to improve the reproducibility of the work that we publish. This form provides structure for consistency and transparency in reporting. For further information on Nature Portfolio policies, see our Editorial Policies and the Editorial Policy Checklist.

Statistics

For all statistical analyses, confirm that the following items are present in the figure legend, table legend, main text, or Methods section.

n/a	Confirmed	
☐	☒	The exact sample size (*n*) for each experimental group/condition, given as a discrete number and unit of measurement
☐	☒	A statement on whether measurements were taken from distinct samples or whether the same sample was measured repeatedly
☐	☒	The statistical test(s) used AND whether they are one- or two-sided<br>*Only common tests should be described solely by name; describe more complex techniques in the Methods section.*
☒	☐	A description of all covariates tested
☐	☒	A description of any assumptions or corrections, such as tests of normality and adjustment for multiple comparisons
☐	☒	A full description of the statistical parameters including central tendency (e.g. means) or other basic estimates (e.g. regression coefficient) AND variation (e.g. standard deviation) or associated estimates of uncertainty (e.g. confidence intervals)
☐	☒	For null hypothesis testing, the test statistic (e.g. *F*, *t*, *r*) with confidence intervals, effect sizes, degrees of freedom and *P* value noted<br>*Give P values as exact values whenever suitable.*
☒	☐	For Bayesian analysis, information on the choice of priors and Markov chain Monte Carlo settings
☒	☐	For hierarchical and complex designs, identification of the appropriate level for tests and full reporting of outcomes
☒	☐	Estimates of effect sizes (e.g. Cohen's *d*, Pearson's *r*), indicating how they were calculated

Our web collection on statistics for biologists contains articles on many of the points above.

Software and code

Policy information about availability of computer code

Data collection

FV31S-SW Viewer software(v2.6, Olympus), Zeiss ZEN software (v3.1, Zeiss) and Nikon NIS-Elements Viewer software (v5.21, Nikon) were used for microscopy.
PANNORAMIC Scanner Software (v3.0, 3DHISTECH) was used for H&E and IHC staining.
Living Image software (v4.5.5, PerkinElmer) was used for in vivo imaging.
BD FACSDiva (v8.0, BD Biosciences) software was used for flow cytometry.
Seahorse Wave Desktop software (v2.6.1.53, Agilent) was used for ECAR and OCR data collection.
Mascot search engine (v2.3; Matrix Science) with the Proteome Discoverer software program (v1.4, ThermoFisher Scientific) has been used for proteomics studies.
Xcalibur software (v4.0.27.10, ThermoFisher Scientific) was used for identification of phosphorylatjon site(s).
TraceFinder software (v5.1, ThermoFisher Scientific) was used for data collection of intermediate metabolites.

Data analysis

All statistical analysis was obtained using the GraphPad Prism software (v9.0) and Microsoft Excel (2019) .
Living Image software (IVIS Imaging Systems v4.5.5, PerkinElmer) was used for analyzing in vivo optical data.
FlowJo software (v10.7.2, BD Biosciences) was used for analyzing flow cytometry data.
Softmax Pro software (v6.3, Molecular Device) was used for protein qualification and enzymatic activity analysis.
Seahorse Wave Desktop software (v2.6.1.53, Agilent) and Microsoft Excel (2019) was used for ECAR and OCR analysis.
Image J (v1.46r, NIH) was used for immunoblotting and immunofluorescence staining analyses.
Caseviewer (v2.2,3DHISTECH) was used for data analysis of H&E and IHC staining.
Mascot search engine (v2.3, Matrix Science) with the Proteome Discoverer software program (v1.4, ThermoFisher Scientific) has been used

for proteomics study analysis.
Xcalibur software (v4.0.27.10, ThermoFisher Scientific) was used for phosphorylatjon site(s) analysis.

For manuscripts utilizing custom algorithms or software that are central to the research but not yet described in published literature, software must be made available to editors and reviewers. We strongly encourage code deposition in a community repository (e.g. GitHub). See the Nature Portfolio guidelines for submitting code & software for further information.

Data

Policy information about availability of data

All manuscripts must include a data availability statement. This statement should provide the following information, where applicable:

- Accession codes, unique identifiers, or web links for publicly available datasets
- A description of any restrictions on data availability
- For clinical datasets or third party data, please ensure that the statement adheres to our policy

All main figures and extended data figures have source data and/or original figures, which have been included in the submission. The proteomics and mass spectrometry data generated in this study have been deposited in the ProteomeXchange database under accession code PXD040380 and PXD040377, respectively. The metabolomics data used in this study have been deposited in the MetaboLights database under accession code MTBLS8218. The processed metabolomics data in this study are provided in the Source Data file. UniProt protein database (EMBL-EBI) was used for protein identification. All other data are available from the corresponding author upon reasonable request.

Research involving human participants, their data, or biological material

Policy information about studies with human participants or human data. See also policy information about sex, gender (identity/presentation), and sexual orientation and race, ethnicity and racism.

Reporting on sex and gender	N/A
Reporting on race, ethnicity, or other socially relevant groupings	N/A
Population characteristics	N/A
Recruitment	N/A
Ethics oversight	N/A

Note that full information on the approval of the study protocol must also be provided in the manuscript.

Field-specific reporting

Please select the one below that is the best fit for your research. If you are not sure, read the appropriate sections before making your selection.

[x] Life sciences [ ] Behavioural & social sciences [ ] Ecological, evolutionary & environmental sciences

For a reference copy of the document with all sections, see nature.com/documents/nr-reporting-summary-flat.pdf

Life sciences study design

All studies must disclose on these points even when the disclosure is negative.

Sample size	Sample sizes were chosen based on our experience with experimental models and previous publications(PMID 30409970; PMID 30842655; PMID 32273511; PMID 35228743) and were sufficient for statistical analysis. Sample numbers were described in the Figure legends. No statistical methods were used to predetermine sample size.
Data exclusions	No data exclusion.
Replication	All replication attempts were successful. Experiments were independently repeated at least three times with similar results.
Randomization	Animals were randomly allocated to each group. Randomization was applied for biochemical and in vitro experiments because there were defined groups. e.g. control or specific shRNA groups.
Blinding	The Mass spectrometry analyses (proteome assay, detection of phosphorylation sites assay and Endman sequencing assay) were performed by technical staffs at the Protein Chemistry and Proteomic Core of Tsinghua University who were blinded to the experimental groups. Influx of intermediate metabolites analyses were performed by the technical staffs at the Metabolomics and Lipidomics Core of Tsinghua University who were blinded to the experimental groups. Cellular and biochemical experiments were not performed in a blinding manner because there were defined groups.

Behavioural & social sciences study design

All studies must disclose on these points even when the disclosure is negative.

Study description	*Briefly describe the study type including whether data are quantitative, qualitative, or mixed-methods (e.g. qualitative cross-sectional, quantitative experimental, mixed-methods case study).*
Research sample	*State the research sample (e.g. Harvard university undergraduates, villagers in rural India) and provide relevant demographic information (e.g. age, sex) and indicate whether the sample is representative. Provide a rationale for the study sample chosen. For studies involving existing datasets, please describe the dataset and source.*
Sampling strategy	*Describe the sampling procedure (e.g. random, snowball, stratified, convenience). Describe the statistical methods that were used to predetermine sample size OR if no sample-size calculation was performed, describe how sample sizes were chosen and provide a rationale for why these sample sizes are sufficient. For qualitative data, please indicate whether data saturation was considered, and what criteria were used to decide that no further sampling was needed.*
Data collection	*Provide details about the data collection procedure, including the instruments or devices used to record the data (e.g. pen and paper, computer, eye tracker, video or audio equipment) whether anyone was present besides the participant(s) and the researcher, and whether the researcher was blind to experimental condition and/or the study hypothesis during data collection.*
Timing	*Indicate the start and stop dates of data collection. If there is a gap between collection periods, state the dates for each sample cohort.*
Data exclusions	*If no data were excluded from the analyses, state so OR if data were excluded, provide the exact number of exclusions and the rationale behind them, indicating whether exclusion criteria were pre-established.*
Non-participation	*State how many participants dropped out/declined participation and the reason(s) given OR provide response rate OR state that no participants dropped out/declined participation.*
Randomization	*If participants were not allocated into experimental groups, state so OR describe how participants were allocated to groups, and if allocation was not random, describe how covariates were controlled.*

Ecological, evolutionary & environmental sciences study design

All studies must disclose on these points even when the disclosure is negative.

Study description	*Briefly describe the study. For quantitative data include treatment factors and interactions, design structure (e.g. factorial, nested, hierarchical), nature and number of experimental units and replicates.*
Research sample	*Describe the research sample (e.g. a group of tagged Passer domesticus, all Stenocereus thurberi within Organ Pipe Cactus National Monument), and provide a rationale for the sample choice. When relevant, describe the organism taxa, source, sex, age range and any manipulations. State what population the sample is meant to represent when applicable. For studies involving existing datasets, describe the data and its source.*
Sampling strategy	*Note the sampling procedure. Describe the statistical methods that were used to predetermine sample size OR if no sample-size calculation was performed, describe how sample sizes were chosen and provide a rationale for why these sample sizes are sufficient.*
Data collection	*Describe the data collection procedure, including who recorded the data and how.*
Timing and spatial scale	*Indicate the start and stop dates of data collection, noting the frequency and periodicity of sampling and providing a rationale for these choices. If there is a gap between collection periods, state the dates for each sample cohort. Specify the spatial scale from which the data are taken*
Data exclusions	*If no data were excluded from the analyses, state so OR if data were excluded, describe the exclusions and the rationale behind them, indicating whether exclusion criteria were pre-established.*
Reproducibility	*Describe the measures taken to verify the reproducibility of experimental findings. For each experiment, note whether any attempts to repeat the experiment failed OR state that all attempts to repeat the experiment were successful.*
Randomization	*Describe how samples/organisms/participants were allocated into groups. If allocation was not random, describe how covariates were controlled. If this is not relevant to your study, explain why.*
Blinding	*Describe the extent of blinding used during data acquisition and analysis. If blinding was not possible, describe why OR explain why blinding was not relevant to your study.*

Did the study involve field work? ☐ Yes ☐ No

Field work, collection and transport

Field conditions	*Describe the study conditions for field work, providing relevant parameters (e.g. temperature, rainfall).*
Location	*State the location of the sampling or experiment, providing relevant parameters (e.g. latitude and longitude, elevation, water depth).*
Access & import/export	*Describe the efforts you have made to access habitats and to collect and import/export your samples in a responsible manner and in compliance with local, national and international laws, noting any permits that were obtained (give the name of the issuing authority, the date of issue, and any identifying information).*
Disturbance	*Describe any disturbance caused by the study and how it was minimized.*

Reporting for specific materials, systems and methods

We require information from authors about some types of materials, experimental systems and methods used in many studies. Here, indicate whether each material, system or method listed is relevant to your study. If you are not sure if a list item applies to your research, read the appropriate section before selecting a response.

Materials & experimental systems

n/a	Involved in the study	
☐	☒	Antibodies
☐	☒	Eukaryotic cell lines
☒	☐	Palaeontology and archaeology
☐	☒	Animals and other organisms
☒	☐	Clinical data
☒	☐	Dual use research of concern
☒	☐	Plants

Methods

n/a	Involved in the study	
☒	☐	ChIP-seq
☐	☒	Flow cytometry
☒	☐	MRI-based neuroimaging

Antibodies

Antibodies used

Antibodies used in this study were purchased from the indicated sources:
anti-Flag (Sigma, F1804; 1:10,000 dilution in immunoblotting; 1:1,000 dilution in immunofluorescence),
anti-HA (Bioeasytech, BE2007; 1:3,000 dilution in immunoblotting; 1:1,000 dilution in immunofluorescence),
anti-GFP (MBL, 598; 1:3,000 dilution in immunoblotting),
anti-panAKT(C67E7) (CST, 4691, 1:1,000 dilution in immunoblotting)
anti-AKT1(C73H10) (CST, 2983; 1:1,000 dilution in immunoblotting),
anti-pAKT T308(C31E5E) (CST, 2965; 1:1,000 dilution in immunoblotting),
anti-pAKT S473 (D9E) (CST, 4060; 1:1,000 dilution in immunoblotting; 1:100 in immunohistochemistry),
anti-COXIV (Proteintech, 11242-1-AP; 1:1,000 dilution in immunoblotting; 1:100 dilution in immunofluorescence),
anti-pAKT substrate RXXS/T(110B7E) (CST, 9614; 1:1,000 dilution in immunoblotting),
anti-ME2 (Proteintech, 24944-1-AP; 1:1,000 dilution in immunoblotting; 1:100 dilution in immunoprecipitation),
anti-ME2 (Proteintech, 67457-1-IG; 1:100 dilution in immunohistochemistry),
anti-ME2 (E1N3F) (CST, 35939; 1:20 dilution in immunogold electronic microscope; 1:400 dilution in immunofluorescence),
anti-GAPDH (Proteintech, 60004-1-Ig; 1:3,000 dilution in immunoblotting; 1:100 in immunoprecipitation),
anti-PFKL (A-6) (Santa Cruz, sc-393713; 1:1,000 dilution in immunoblotting; 1:100 in immunoprecipitation),
anti-PKM2 (D78A4) (CST, 4053; 1:1,000 dilution in immunoblotting; 1:100 in immunoprecipitation),
anti-LDH-A (Proteintech, 19981-1-AP; 1:3,000 dilution in immunoblotting; 1:100 in immunoprecipitation),
anti-GSK3β(1F7) (Santa Cruz, sc-53931; 1:1,000 dilution in immunoblotting),
anti-pGSK3β-S9 (CST, 9336; 1:1,000 dilution in immunoblotting),
anti-p-p70S6K-T389 (108D2) (CST, 9234; 1:1,1000 dilution in immunoblotting),
anti-p70S6K (CST, 9202; 1:1,1000 dilution in immunoblotting),
anti-pS6-235/236 (D57.2.2E) (CST, 4858; 1:1,1000 dilution in immunoblotting),
anti-S6 (CST, 2217; 1:1,1000 dilution in immunoblotting),
anti-β-Actin (Bioeasytech, BE0037; 1:5,000 dilution in immunoblotting),
anti-GST (Bioeasytech, BE2013; 1:5,000 dilution in immunoblotting),
anti-Tubulin (Bioeasytech, BE0031; 1:3,000 dilution in immunoblotting),
Normal mouse IgG (Santa Cruz, sc2025; 1:100 dilution in immunoprecipitation),
Normal Rabbit IgG (CST, 2729; 1:100 dilution in immunoprecipitation),
Goat anti-rabbit IgG-HRP (Bioeasytech, BE0101, 1:5,000 dilution in immunoblotting),
Goat anti-mouse IgG-HRP (Bioeasytech, BE0102, 1:5,000 dilution in immunoblotting),
Alexa Fluor® 488 Goat anti-Rabbit IgG (H+L) (ThermoFisher Scientific, A11008; 1:1,000 dilution in immunofluorescence),
Alexa Fluor® 594 Goat Anti-Mouse (ThermoFisher Scientific, R37115; 1:40 dilution in immunofluorescence),

Goat anti-Mouse IgG (H+L) Cross-Adsorbed Secondary Antibody, Alexa Fluor® 647 conjugate (ThermoFisher Scientific, A32728; 1:1,000 dilution in immunofluorescence),
Goat anti-Rabbit IgG (H+L) Cross-Adsorbed Secondary Antibody, Alexa Fluor® 647 conjugate (ThermoFisher Scientific, A-21246; 1:1,000 dilution in immunofluorescence).
The custom-designed polyclonal antibodies listed as followings were in cooperation with Abclonal Technology (Wuhan, China): anti-phospho-ME2fl Ser9 (p-ME2fl-S9, antigen peptide: C-SRLRVV(S-p)TT ;1:1,000 dilution in immunoblotting),
anti-N-ME2fl (anti-N-ME2fl, antigen peptide: SRLRVVSTTCTLACRH; 1:1,000 dilution in immunoblotting),
Colloidal Gold AffiniPure Goat Anti-Rabbit IgG (H+L) (Jackson ImmunoResearch Laboratories, 111-195-144; 1:50 dilution in electron microscopic immunogold staining)

Validation

The custom-designed polyclonal antibodies for immunoblotting and IHC analyses were validated by dot blot assays.
anti-phospho-ME2fl Ser9 (p-ME2fl-S9): Fig.2j, 3f, 3g, 7f, Extended Data Fig. 5f
anti-N-ME2fl (anti-N-ME2fl): Fig.7f, Extended Data Fig. 9a-e
Other antibodies used in this work were purchased from companies, and validated by the manufacturers and by extensive use in published work.
anti-Flag antibody: https://www.sigmaaldrich.cn/CN/zh/product/sigma/f1804
anti-HA antibody: http://bioeasytech.com/product/2385.html?goods_id=4370
anti-GFP antibody: https://www.mblbio.com/bio/g/dtl/A/?pcd=598
anti-panAKT(C67E7): https://www.cellsignal.cn/products/primary-antibodies/akt-pan-c67e7-rabbit-mab/4691
anti-AKT1 antibody: https://www.cellsignal.com/products/primary-antibodies/akt1-c73h10-rabbit-mab/2938
anti-pAKT T308(C31E5E) antibody: https://www.cellsignal.cn/products/primary-antibodies/phospho-akt-thr308-c31e5e-rabbit-mab/2965
anti-pAKT S473 antibody: https://www.cellsignal.cn/products/primary-antibodies/phospho-akt-ser473-d9e-xp-rabbit-mab/4060
anti-COXIV: https://www.ptgcn.com/products/COX4I1-Antibody-11242-1-AP.htm
anti-pAKT substrate RXXS/T: https://www.cellsignal.cn/products/primary-antibodies/phospho-akt-substrate-rxxs-t-110b7e-rabbit-mab/9614
anti-ME2: https://www.ptgcn.com/products/ME2-Antibody-24944-1-AP.htm
anti-ME2: https://www.ptgcn.com/products/ME2-Antibody-67457-1-Ig.htm
anti-ME2: https://www.cellsignal.cn/product/productDetail.jsp?productId=35939
anti-GAPDH: https://www.ptgcn.com/products/GAPDH-Antibody-60004-1-Ig.htm
anti-PFKL(A-6): https://www.scbt.com/zh/p/pfkl-antibody-a-6
anti-PKM2(D78A4): https://www.cellsignal.cn/products/primary-antibodies/pkm2-d78a4-xp-rabbit-mab/4053
anti-LDH-A: https://www.ptgcn.com/products/LDHA-Specific-Antibody-19987-1-AP.htm
anti-GSK3β(1F7): https://www.scbt.com/p/gsk-3beta-antibody-1f7
anti-pGSK3β-S9: https://www.cellsignal.cn/products/primary-antibodies/phospho-gsk-3b-ser9-antibody/9336
anti-p-p70S6K-T389(108D2): https://www.cellsignal.cn/products/primary-antibodies/phospho-p70-s6-kinase-thr389-108d2-rabbit-mab/9234
anti-p70S6K: https://www.cellsignal.cn/products/primary-antibodies/p70-s6-kinase-antibody/9202
anti-pS6-235/236(D57.2.2E): https://www.cellsignal.cn/products/primary-antibodies/phospho-s6-ribosomal-protein-ser235-236-d57-2-2e-xp-rabbit-mab/4858
anti-S6(5G10): https://www.cellsignal.cn/products/primary-antibodies/s6-ribosomal-protein-5g10-rabbit-mab/2217
anti-β-Actin: http://www.bioeasytech.com/product/2382.html?goods_id=4300
anti-GST: http://www.bioeasytech.com/product/2390.html?goods_id=4375
anti-Tubulin: http://www.bioeasytech.com/product/2369.html?goods_id=4287
Normal mouse IgG: https://www.scbt.com/p/normal-mouse-igg
Normal Rabbit IgG: https://www.cellsignal.cn/products/primary-antibodies/normal-rabbit-igg/2729
Goat anti-rabbit IgG-HRP: http://www.bioeasytech.com/product/2901.html?goods_id=5786
Goat anti-mouse IgG-HRP: http://www.bioeasytech.com/product/2907.html?goods_id=5794
Alexa Fluor® 488 Goat anti-Rabbit IgG (H+L): https://www.thermofisher.cn/cn/zh/antibody/product/Goat-anti-Rabbit-IgG-H-L-Cross-Adsorbed-Secondary-Antibody-Polyclonal/A-11008
Alexa Fluor® 594 Goat Anti-Mouse: https://www.thermofisher.cn/cn/zh/antibody/product/Donkey-anti-Mouse-IgG-H-L-Secondary-Antibody-Polyclonal/R37115
Goat anti-Mouse IgG (H+L) Cross-Adsorbed Secondary Antibody, Alexa Fluor® 647 conjugate: https://www.thermofisher.cn/cn/zh/antibody/product/Goat-anti-Mouse-IgG-H-L-Highly-Cross-Adsorbed-Secondary-Antibody-Polyclonal/A32728
Goat anti-Rabbit IgG (H+L) Cross-Adsorbed Secondary Antibody, Alexa Fluor® 647 conjugate: https://www.thermofisher.cn/cn/zh/antibody/product/Goat-anti-Rabbit-IgG-H-L-Cross-Adsorbed-Secondary-Antibody-Polyclonal/A-21246
Colloidal Gold AffiniPure Goat Anti-Rabbit IgG (H+L): https://www.jacksonimmuno.com/catalog/products/111-195-144

Eukaryotic cell lines

Policy information about cell lines and Sex and Gender in Research

Cell line source(s)

HEK293T cells, human hepatoma HepG2 cells, human osteosarcoma U2OS cells, human colon cancer HCT116 cells, human lung carcinoma A549 cells and PC9 cells, human non-small cell lung cancer H1299 cells, human prostate adenocarcinoma PC3 cells, human glioblastoma U87MG cells and human breast cancer cell line MDA-MB-231 were purchased from ATCC.

Authentication

To authenticate cell lines, we used short tandem repeat (STR) profling method as exhaustively described by Dr. Asadi Jahanbakhsh and his/her colleagues (Khosravi Ayyoob et al, Tumor Biol.(2016) 37:3197-3204).

Mycoplasma contamination	All cell lines were subjected to examination of mycoplasma contamination by using a Mycoplasma Detection Kit purchased from Lonza (Catalog number: LTO7). All cell lines for experiment were tested negative for mycoplasma contamination.
Commonly misidentified lines (See ICLAC register)	None of the cell lines used in this work was listed as "Misidentified Cell Line" in the ICLAC database.

Palaeontology and Archaeology

Specimen provenance	*Provide provenance information for specimens and describe permits that were obtained for the work (including the name of the issuing authority, the date of issue, and any identifying information). Permits should encompass collection and, where applicable, export.*
Specimen deposition	*Indicate where the specimens have been deposited to permit free access by other researchers.*
Dating methods	*If new dates are provided, describe how they were obtained (e.g. collection, storage, sample pretreatment and measurement), where they were obtained (i.e. lab name), the calibration program and the protocol for quality assurance OR state that no new dates are provided.*

☐ Tick this box to confirm that the raw and calibrated dates are available in the paper or in Supplementary Information.

Ethics oversight	*Identify the organization(s) that approved or provided guidance on the study protocol, OR state that no ethical approval or guidance was required and explain why not.*

Note that full information on the approval of the study protocol must also be provided in the manuscript.

Animals and other research organisms

Policy information about studies involving animals; ARRIVE guidelines recommended for reporting animal research, and Sex and Gender in Research

Laboratory animals	4- to 5-week-old male athymic Balb-c nu/nu male mice were purchased from Huafukang Laboratory Animal Technology (Beijing, China) for xenograft experiment. The 10-week old wild type Pb-Cre-;PtenL/L and Pten condition knock-out Pb-Cre+;PtenL/L mice were kindly gifted by Prof. Hong Wu (Peking University, Beijing). All mice were housed in isolated ventilated cages (maxima six mice per cage) barrier facility at Tsinghua University. The mice were maintained on a 12/12-hour light/dark cycle, 22-26'C, 50% humidity with sterile pellet food and water ad libitum.
Wild animals	No wild animals were used in this study.
Reporting on sex	All animals used in this study were male.
Field-collected samples	This study did not involve these samples.
Ethics oversight	All animals were treated and used with the approval of The Animal Care and Use Committee of Tsinghua University. All the tumors' sizes didn't exceed the TUACUC-approved maximum size (10% of mouse weight, 1.8-2.0g usually). All animal were kept according to guidelines and regulations approved by the Tsinghua University Animal Care and Use Committee.

Note that full information on the approval of the study protocol must also be provided in the manuscript.

Clinical data

Policy information about clinical studies

All manuscripts should comply with the ICMJEguidelines for publication of clinical research and a completedCONSORT checklist must be included with all submissions.

Clinical trial registration	*Provide the trial registration number from ClinicalTrials.gov or an equivalent agency.*
Study protocol	*Note where the full trial protocol can be accessed OR if not available, explain why.*
Data collection	*Describe the settings and locales of data collection, noting the time periods of recruitment and data collection.*
Outcomes	*Describe how you pre-defined primary and secondary outcome measures and how you assessed these measures.*

Dual use research of concern

Policy information about dual use research of concern

Hazards

Could the accidental, deliberate or reckless misuse of agents or technologies generated in the work, or the application of information presented in the manuscript, pose a threat to:

No	Yes	
x		Public health
x		National security
x		Crops and/or livestock
x		Ecosystems
x		Any other significant area

Experiments of concern

Does the work involve any of these experiments of concern:

No	Yes	
x		Demonstrate how to render a vaccine ineffective
x		Confer resistance to therapeutically useful antibiotics or antiviral agents
x		Enhance the virulence of a pathogen or render a nonpathogen virulent
x		Increase transmissibility of a pathogen
x		Alter the host range of a pathogen
x		Enable evasion of diagnostic/detection modalities
x		Enable the weaponization of a biological agent or toxin
x		Any other potentially harmful combination of experiments and agents

Plants

Seed stocks	*Report on the source of all seed stocks or other plant material used. If applicable, state the seed stock centre and catalogue number. If plant specimens were collected from the field, describe the collection location, date and sampling procedures.*
Novel plant genotypes	*Describe the methods by which all novel plant genotypes were produced. This includes those generated by transgenic approaches, gene editing, chemical/radiation-based mutagenesis and hybridization. For transgenic lines, describe the transformation method, the number of independent lines analyzed and the generation upon which experiments were performed. For gene-edited lines, describe the editor used, the endogenous sequence targeted for editing, the targeting guide RNA sequence (if applicable) and how the editor was applied.*
Authentication	*Describe any authentication procedures for each seed stock used or novel genotype generated. Describe any experiments used to assess the effect of a mutation and, where applicable, how potential secondary effects (e.g. second site T-DNA insertions, mosiacism, off-target gene editing) were examined.*

ChIP-seq

Data deposition

- [ ] Confirm that both raw and final processed data have been deposited in a public database such as GEO.
- [ ] Confirm that you have deposited or provided access to graph files (e.g. BED files) for the called peaks.

Data access links<br>*May remain private before publication.*	*For "Initial submission" or "Revised version" documents, provide reviewer access links. For your "Final submission" document, provide a link to the deposited data.*
Files in database submission	*Provide a list of all files available in the database submission.*
Genome browser session<br>(e.g. UCSC)	*Provide a link to an anonymized genome browser session for "Initial submission" and "Revised version" documents only, to enable peer review. Write "no longer applicable" for "Final submission" documents.*

Methodology

Replicates	*Describe the experimental replicates, specifying number, type and replicate agreement.*
Sequencing depth	*Describe the sequencing depth for each experiment, providing the total number of reads, uniquely mapped reads, length of reads and whether they were paired- or single-end.*
Antibodies	*Describe the antibodies used for the ChIP-seq experiments; as applicable, provide supplier name, catalog number, clone name, and lot number.*

Peak calling parameters	*Specify the command line program and parameters used for read mapping and peak calling, including the ChIP, control and index files used.*
Data quality	*Describe the methods used to ensure data quality in full detail, including how many peaks are at FDR 5% and above 5-fold enrichment.*
Software	*Describe the software used to collect and analyze the ChIP-seq data. For custom code that has been deposited into a community repository, provide accession details.*

Flow Cytometry

Plots

Confirm that:

- [x] The axis labels state the marker and fluorochrome used (e.g. CD4-FITC).
- [x] The axis scales are clearly visible. Include numbers along axes only for bottom left plot of group (a 'group' is an analysis of identical markers).
- [x] All plots are contour plots with outliers or pseudocolor plots.
- [x] A numerical value for number of cells or percentage (with statistics) is provided.

Methodology

Sample preparation	For ROS measurement, cells were incubated at 37℃ ’C ℃ for 30 min in PBS containing 10μM 2’,7’-dichlorodihydrofluorescein diacetate (H2-DCFDA). After incubation, cells were digested and re-suspended in PBS. Fluorescence was measured using BD LSRFortessa SORP.<br>SgRNA and donor plasmids were co-transfected with a 1:1 ratio (1 μg sgRNA and 1 μg donor plasmids) into MDA-MB-231 cells at 70% confluence in a 6 cm cell culture plate. 24 h after transfection, cells were trypinzed and GFP positive cells were sorted by BD FACSAria SORP for GFP positive single cell and seeded in 96-well plates.
Instrument	BD LSRFortessa SORP was used for ROS measurement and BD FACSAria SORP for cell sorting.
Software	BD FACSDiva (v8.0, BD Biosciences) software was used for flow cytometry data collection and FlowJo software (v10.7.2, BD Biosciences) was used for analyzing flow cytometry data.
Cell population abundance	For ROS measurement, the purity was determined by the percentage of FITC positive cells; For GFP positive-sorted cells, the purity was determined by the percentage of GFP positive cells during sorting.
Gating strategy	Gate was applied excluding the GFP-negative cells and including the GFP-positive cells only. For DCF staining, positive DCF was defined for cells incubated with DCF.

- [x] Tick this box to confirm that a figure exemplifying the gating strategy is provided in the Supplementary Information.

Magnetic resonance imaging

Experimental design

Design type	*Indicate task or resting state; event-related or block design.*
Design specifications	*Specify the number of blocks, trials or experimental units per session and/or subject, and specify the length of each trial or block (if trials are blocked) and interval between trials.*
Behavioral performance measures	*State number and/or type of variables recorded (e.g. correct button press, response time) and what statistics were used to establish that the subjects were performing the task as expected (e.g. mean, range, and/or standard deviation across subjects).*

Acquisition

Imaging type(s)	*Specify: functional, structural, diffusion, perfusion.*
Field strength	*Specify in Tesla*
Sequence & imaging parameters	*Specify the pulse sequence type (gradient echo, spin echo, etc.), imaging type (EPI, spiral, etc.), field of view, matrix size, slice thickness, orientation and TE/TR/flip angle.*
Area of acquisition	*State whether a whole brain scan was used OR define the area of acquisition, describing how the region was determined.*

Diffusion MRI ☐ Used ☐ Not used

Preprocessing

Preprocessing software	*Provide detail on software version and revision number and on specific parameters (model/functions, brain extraction, segmentation, smoothing kernel size, etc.).*
Normalization	*If data were normalized/standardized, describe the approach(es): specify linear or non-linear and define image types used for transformation OR indicate that data were not normalized and explain rationale for lack of normalization.*
Normalization template	*Describe the template used for normalization/transformation, specifying subject space or group standardized space (e.g. original Talairach, MNI305, ICBM152) OR indicate that the data were not normalized.*
Noise and artifact removal	*Describe your procedure(s) for artifact and structured noise removal, specifying motion parameters, tissue signals and physiological signals (heart rate, respiration).*
Volume censoring	*Define your software and/or method and criteria for volume censoring, and state the extent of such censoring.*

Statistical modeling & inference

Model type and settings	*Specify type (mass univariate, multivariate, RSA, predictive, etc.) and describe essential details of the model at the first and second levels (e.g. fixed, random or mixed effects; drift or auto-correlation).*
Effect(s) tested	*Define precise effect in terms of the task or stimulus conditions instead of psychological concepts and indicate whether ANOVA or factorial designs were used.*

Specify type of analysis: ☐ Whole brain ☐ ROI-based ☐ Both

Statistic type for inference<br>(See Eklund et al. 2016)	*Specify voxel-wise or cluster-wise and report all relevant parameters for cluster-wise methods.*
Correction	*Describe the type of correction and how it is obtained for multiple comparisons (e.g. FWE, FDR, permutation or Monte Carlo).*

Models & analysis

n/a	Involved in the study
☐	☐ Functional and/or effective connectivity
☐	☐ Graph analysis
☐	☐ Multivariate modeling or predictive analysis

Functional and/or effective connectivity	*Report the measures of dependence used and the model details (e.g. Pearson correlation, partial correlation, mutual information).*
Graph analysis	*Report the dependent variable and connectivity measure, specifying weighted graph or binarized graph, subject- or group-level, and the global and/or node summaries used (e.g. clustering coefficient, efficiency, etc.).*
Multivariate modeling and predictive analysis	*Specify independent variables, features extraction and dimension reduction, model, training and evaluation metrics.*
