# Supplementary material for: AKT1 phosphorylation of cytoplasmic ME2 induces a metabolic switch to glycolysis for tumorigenesis
Source: Nat Commun. 2024 Jan 23;15:686. doi: 10.1038/s41467-024-44772-8 (PMC10805786; doi:10.1038/s41467-024-44772-8)
Supplement: Supplementary file 3 — Description of Additional Supplementary Files [file 41467_2024_44772_MOESM3_ESM.pdf]

Description of Additional Supplementary Files

File name: Supplementary Data 1:
Description: Related to Table 1. List of proteins obtained by mass spectrometry analysis of ME2flS9D-3'Flag immunoprecipitants. HEK293T cells expressing ME2flS9D-3'Flag or vector control were lysed, immunoprecipitated with anti-Flag antibody and analyzed by high sensitivity LC-MS/MS using an Orbitrap Elite mass spectrometer (ThermoFisher Scientific). Fragment spectra were searched against the UniProt protein database for protein identification. The glycolytic enzymes that may interact with phosphorylated ME2fl and their mass spectrometry results are shown.

File name: Supplementary Data 2:
Description: List of primers used to generate ME2fl-related constructs in the paper.
